# Engineering 2D Multienzyme‐Mimicking Pyroptosis Inducers for Ultrasound‐Augmented Catalytic Tumor Nanotherapy

**DOI:** 10.1002/advs.202301279

**Published:** 2023-06-23

**Authors:** Xinran Song, Hui Huang, Lili Xia, Wencong Jia, Shaoling Yang, Chenglong Wang, Yu Chen

**Affiliations:** ^1^ School of Environmental and Chemical Engineering Shanghai University Shanghai 200444 P. R. China; ^2^ Department of Ultrasound Medicine Shanghai Eighth People's Hospital Shanghai 200235 P. R. China; ^3^ Department of Orthopedic Surgery XinHua Hospital Affiliated with Shanghai Jiaotong University School of Medicine Shanghai 200082 P. R. China

**Keywords:** 2D nanosheets, catalytic therapy, multienzyme‐mimicking, pyroptosis, ultrasound therapy

## Abstract

Overcoming apoptosis resistance is necessary to ensure an effective cancer treatment; however, it is currently very difficult to achieve. A desirable alternative for cancer treatment is the targeted activation of pyroptosis, a unique type of programmed cell death. However, the pyroptosis inducers that are efficient for cancer therapy are limited. This work reports the engineering of 2D NiCoO*_x_* nanosheets as inducers of the production of harmful reactive oxygen species (ROS), which promote intense cell pyroptosis, and that can be applied to ultrasound (US)‐augmented catalytic tumor nanotherapy. The main therapeutic task is carried out by the 2D NiCoO*_x_* nanosheets, which have four multienzyme‐mimicking activities: peroxidase‐ (POD), oxidase‐ (OXD), glutathione peroxidase‐ (GPx), and catalase‐ (CAT) mimicking activities. These activities induce the reversal of the hypoxic microenvironment, endogenous glutathione depletion, and a continuous ROS output. The ROS‐induced pyroptosis process is carried out via the ROS‐NLRP3‐GSDMD pathway, and the exogenous US activation boosts the multienzyme‐mimicking activities and favors the incremental ROS generation, thus inducing mitochondrial dysfunction. The anti‐cancer experimental results support the dominance of NiCoO*_x_* nanosheet‐induced pyroptosis. This work expands on the biomedical applications of engineering 2D materials for US‐augmented catalytic breast cancer nanotherapy and deepens the understanding of the multienzyme activities of nanomaterials.

## Graphical abstract

The 2D multienzyme‐mimicking NiCoO*_x_* nanosheets as proptosis inducers have been developed to increase the rate of deleterious reactive oxygen species generation for the ultrasound‐augmented catalytic nanotherapy of breast cancer.

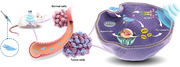

## 1 Introduction

According to the laws of enzyme kinetics under physiological conditions, nanoenzymes are nanomaterials that can catalyze specific chemical reactions and that have enzyme‐mimicking properties.^[^ ^1^ ^]^ Nanoenzymes are similar to natural enzymes in that they share some nanomaterial properties, such as high stability, low preparation costs, straightforward production and purification processes, high catalytic activity, and the capacity to speed up biochemical reactions.^[^ ^2^ ^]^ These nanoenzymes also offer exceptional advantages for medicinal applications, particularly for cancer treatment.^[^ ^3^ ^]^ Representatively, nanozymes with peroxidase (POD)‐mimicking and oxidase (OXD)‐like catalytic capabilities can catalyze the disintegration of hydrogen peroxide (H_2_O_2_) or oxygen (O_2_) and generate highly toxic ROS that can induce the apoptosis or necrosis of cancer cells.^[^ ^4^ ^]^ Thus, the application potential of nanoenzymes for ROS‐mediated tumor‐nanocatalytic treatment is promising.^[^ ^5^ ^]^ Unfortunately, the highly complex tumor microenvironment (TME) influences the effect of the nanoenzyme‐enabled catalytic procedures, such as hypoxia, mildly acidic environment, and overexpression of H_2_O_2_ and glutathione (GSH). To overcome these limitations, H_2_O_2_‐responsive nanoenzymes with dual‐ or multienzyme‐mimicking activities have been explored with broad interest. Besides the above two types of enzyme activities, the glutathione peroxidase (GPx)‐ and catalase (CAT)‐like activities also play important roles. In recent years, nanoenzymes that catalyze reactions of multivalent metal ions (such as Fe^2+/3+^,^[^ ^6^ ^]^ Co^2+/3+^,^[^ ^7^ ^]^ Sn^2+^/Sn^4+^,^[^ ^8^ ^]^ Ni^2+/3+^,^[^ ^9^ ^]^ among others) mimic the function of natural enzymes and play a significant role in regulating the TME, thus enhancing their therapeutic effect. CoFe_2_O_4_ nanoflowers can act as POD‐ and CAT‐mimicking nanoenzymes to catalyze the decomposition of H_2_O_2_ into hydroxyl radicals (•OH) and O_2_ for sonodynamic therapy (SDT) and chemodynamic therapy (CDT).^[^ ^10^ ^]^ Zhang and co‐workers prepared MnFe_2_O_4_ nanoenzymes with GPx‐ and CAT‐like activities that can overcome tumor hypoxia and consume GSH by continuously catalyzing H_2_O_2_ to generate O_2_, to achieve a better therapeutic effect.^[^ ^11^ ^]^ However, nanozymes that concurrently have POD‐, OXD‐, CAT‐, and GPx‐mimicking activities under weak acidic conditions have rarely been explored.

At present, nanoenzyme‐induced cell death has been applied to multiple processes, including apoptosis,^[^ ^12^ ^]^ ferroptosis,^[^ ^13^ ^]^ pyroptosis,^[^ ^14^ ^]^ and so on. Pyroptosis is a form of necrosis that regulates cell death and is performed by the gasdermin (GSDM) family.^[^ ^15^ ^]^ Although GSDMs are expressed in an inactive form, some proteinases activate them via proteolysis. Cleaved gasdermin D (GSDMD) releases the N‐terminal fragment (N‐GSDMD), which forms membrane pores, and then releases pro‐inflammatory molecules into the extracellular environment, thus triggering inflammation and an immunological reaction.^[^ ^16^ ^]^ Ultrasound (US), which is a non‐invasive sound wave that can penetrate deep tissues, possesses acoustic cavitation effects.^[^ ^17^ ^]^ Therefore, the mass diffusion of the nanoenzyme can be improved through US, which significantly improves the enzyme‐like activity and corresponding reaction rates.^[^ ^18^ ^]^ Therefore, appropriately integrating the US‐enhanced enzyme‐like activities into one nanoenzyme can effectively inhibit tumor growth and recurrence.^[^ ^19^ ^]^ Overall, pyroptotic cell death is caused by the activation of multiple cellular protein complexes called inflammasomes; for example, the nucleotide binding oligomerization domain‐like receptor protein 3 (NLRP3) inflammasome acts as a regulatory factor of pyroptosis.^[^ ^20^ ^]^ The activated NLRP3 inflammasomes then transform protoproteinase‐1 into caspase‐1. In contrast with apoptosis, pyroptosis and ferroptosis are immunogenic programmed cell death (PCD) processes, constituting powerful anti‐cancer strategies because they can initiate antitumor immune responses by delivering adequate cytokines and damage‐associated molecular patterns (DAMPs).^[^ ^21^ ^]^ US‐induced pyroptosis can maximize the significant control of tumor growth, thus generating a strong and lasting antitumor response.

**Scheme 1:**
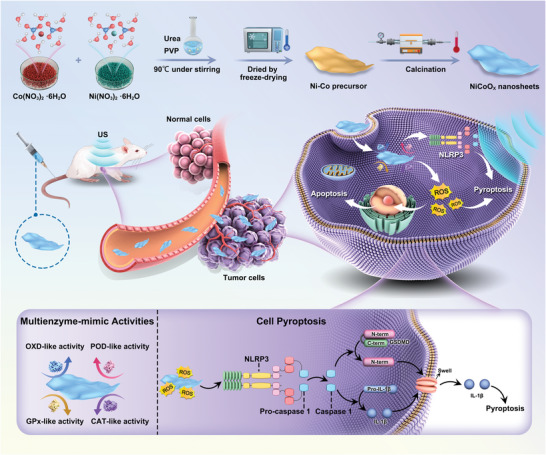
a) The scheme of the synthetic production of the 2D NiCoO*_x_* nanosheets. b) Schematic illustration of the underlying mechanism of how the 2D NiCoO*_x_* nanosheets lead to US‐augmented cell pyroptosis and catalytic tumor nanotherapy.

2D nanomaterials possess exceptional intrinsic characteristics, such as an ultrathin layer, a unique nanosheet structure and surface chemistry, a high surface area and quantum size effect.^[^ ^22^ ^]^ In particular, 2D nanomaterials have become a promising nanoplatform for various biomedical applications due to their specific physical and chemical properties and high drug loading capacity.^[^ ^23^ ^]^ By taking advantage of the interactions between the TME and 2D nanomaterials, an efficient TME‐targeted tumor treatment can be actualized by developing TME‐responsive 2D nanomaterials, which would enable the application of 2D nanomaterials to catalytic medicine and to efficient tumor treatments. To demonstrate our points, herein, morphology‐controlled 2D NiCoO*_x_* nanozymes were successfully constructed using the hard template method.^[^ ^24^ ^]^ The 2D NiCoO*_x_* nanosheets act as multienzyme‐mimicking pyroptosis inducers for US‐augmented catalytic breast cancer nanotherapy without complicated side effects (**Scheme** **1**). The strong catalytic activity of 2D NiCoO*_x_* nanozymes in acidic conditions and upon US simulation was further enhanced to achieve a desirable US‐enhanced effect and pyrolysis‐inducing effects for promoting cancer treatment. Furthermore, the intrinsic Ni^2+^/Ni^3+^ and Co^2+^/Co^3+^ redox couple can produce highly toxic ROS via enzyme‐like activity reactions, thus enhancing its antitumor efficiency. The ROS‐NLRP3‐GSDMD pathway was progressively demonstrated to induce pyroptosis based on the results of transcriptome sequencing, western blot analysis, and enzyme‐linked immunosorbent assays. More importantly, this research provides a new perspective on the design of biocompatible and efficient nanoenzymes for catalyzing cascade cancer therapy and pyroptosis with low side effects. Therefore, this study is highly expected to open a door for tumor treatment and ignite the further exploration and application of nanoenzymes to diverse tumor nanotherapies.

## 2 Results and Discussion

### 2.1 Synthesis and Characterization of the 2D NiCoO*_x_* Nanosheets

**Figure 1:**
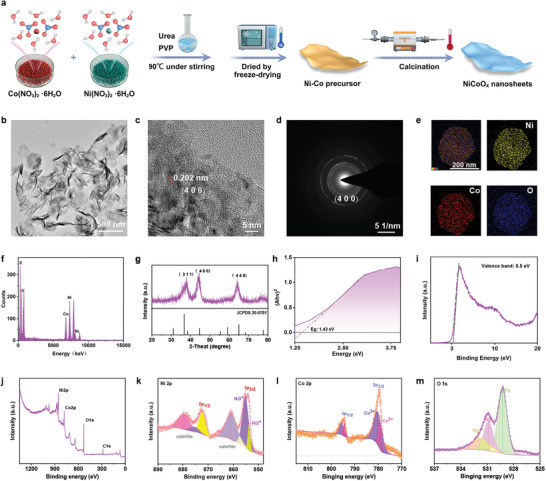
Synthesis and characterization of the 2D NiCoO*_x_* nanosheets. a) The scheme of the synthetic production of the 2D NiCoO*_x_* nanosheets. b) TEM image of the 2D NiCoO*_x_* nanosheets. c) HRTEM image and d) the corresponding selected area electron diffraction pattern of the 2D NiCoO*_x_* nanosheets. e) Corresponding area‐elemental mapping of the 2D NiCoO*_x_* nanosheets. f) Energy‐dispersive spectrometer (EDS) spectrum. g) XRD pattern of the 2D NiCoO*_x_* nanosheets. h) Energy bandgap and i) valence XPS spectra of the 2D NiCoO*_x_* nanosheets. j) XPS spectrum of the 2D NiCoO*_x_* nanosheets. High‐resolution k) Ni 2p, l) Co 2p, and m) O 1s XPS data obtained from the 2D NiCoO*_x_* nanosheets.

The detailed process of the synthetic production of the 2D NiCoO*_x_* nanosheets is displayed in **Figure** **1**a. First, the 2D NiCoO_x_ nanosheets were fabricated using a three‐step synthesis process, including the reaction of Co(NO_3_)_2_·6H_2_O with Ni(NO_3_)_2_·6H_2_O, the addition of hydrophilic polyvinylpyrrolidone (PVP) and urea, and the lyophilization and calcination of the Ni‐Co precursor.^[^ ^24^ ^]^ The morphology of the 2D NiCoO*_x_* nanosheets according to the transmission electron microscopy (TEM) observations is presented in Figure 1b. The 2D NiCoO*_x_* nanosheets have an ultrathin sheet‐shaped structure.^[^ ^24^ ^]^ The TEM image of the 2D NiCoO*_x_* nanosheets shows that they exhibit high dispersibility, with an average particle size of 178 nm, according to the statistical analysis (Figure S1, Supporting Information). The lattice distance of 0.202 nm corresponds to the (400) diffraction plane of the 2D NiCoO*_x_* sheets, as observed from the high‐resolution TEM (HRTEM) images (Figure 1c,d). As shown in Figure 1e,f, X‐ray energy‐dispersive spectrometer (EDS) analysis and the corresponding elemental mapping of the 2D NiCoO*_x_* nanosheets show the coexistence of the Ni, Co, and O elements, demonstrating the successful fabrication of the 2D NiCoO*_x_* nanosheets. Moreover, X‐ray diffraction (XRD) analysis was performed to determine the components, purity, and crystal structure of the fabricated 2D NiCoO*_x_* nanosheets (Figure 1g), in which the characteristic diffraction peaks were found to be well indexed to the standard structure. The pure 2D NiCoO*_x_* nanosheets featured three peaks at 2*θ* values of 38.4°, 44.62°, and 64.98°, which were indexed to the (311), (400), and (440) planes, respectively, and were consistent with the standard values corresponding to NiCo_2_O_4_ (JCPDS 20–0781). The band gap value of the 2D NiCoO*_x_* sheets recorded by the derivative Tauc's plot was around 1.43 eV (Figure 1h). Furthermore, the X‐ray photoelectron spectroscopy (XPS) valence‐band spectrum was utilized to demonstrate the location of the valence band, which was located at 0.5 eV (Figure 1i). Therefore, the ROS generation capability is likely to be induced by US radiation.

Furthermore, to investigate the mixed valence of the components in the NiCoO*_x_* nanosheets, the XPS spectroscopy spectrum of the 2D NiCoO*_x_* nanosheets was recorded. The XPS survey spectrum shows that the nanosheets are composed of the Ni, Co, and O elements (Figure 1j). The analysis of Ni 2p and Co 2p was conducted using high‐resolution XPS (Figures 1k,l). The Ni 2p spectrum (Figure 1k) can be fitted to two shakeup satellites and two prominent 2p_3/2_ and 2p_1/2_ spin‐orbit peaks. In detail, the Ni 2p_3/2_ peak is composed of Ni^2+^ (853.6 eV)/Ni^3+^ (855.5 eV).^[^ ^24^ ^]^ The high‐resolution Co 2p spectrum can also be fitted to two satellites and two spin‐orbit peaks with binding energies, presented in the valence state of Co^2+^ (779.3 eV)/Co^3+^ (781.2 eV), respectively (Figure 1l). The existence of the Ni^2+^/Ni^3+^ and Co^2+^/Co^3+^ redox couple provides tremendous potential for the POD‐, CAT‐, OXD‐, and GPx‐like multienzyme‐mimicking activities. Moreover, the spectrum in Figure 1m shows three main peaks at 529.5, 531.1, and 532 eV, which are derived from O 1s and are assigned to metal‐oxygen bonds, surface hydroxyl groups, or defects with low oxygen coordination states, and adsorbed oxygen, respectively.^[^ ^24^ ^]^

### 2.2 Enzymatic Activities of the 2D NiCoO*_x_* Nanosheets

**Figure 2:**
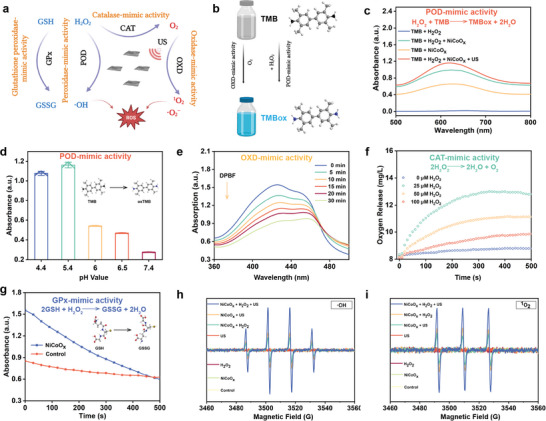
Multienzyme activities of the 2D NiCoO*_x_* nanosheets. a) Catalytic mechanism of the 2D NiCoO*_x_* nanosheets. b) Schematic diagram of the POD‐mimicking catalytic process of the 2D NiCoO*_x_* nanosheets. c) POD‐mimicking activity of the 2D NiCoO*_x_* nanosheets, as tested using a TMB probe with or without NiCoO*_x_*, H_2_O_2_, and US. d) The UV–vis absorbance alterations at 652 nm at different pH values (4.4, 5.4, 6.0, 6.5, and 7.4) originating from TMB oxidation. e) The time‐dependent oxidation of the 2D NiCoO*_x_* nanosheets using DPBF as the trapping agent for demonstrating the production of ^1^O_2_, which is enhanced under US irradiation. f) The CAT‐mimicking activity of 2D NiCoO*_x_*, as determined by measuring the dissolved O_2_ in the presence of different concentrations of H_2_O_2_ (0, 25, 50, and 100 µm). g) GPx‐mimicking activity of the 2D NiCoO*_x_* nanosheets as measured based on the UV–vis absorbance alterations at 340 nm using a GPx assay kit. h) The ESR spectra of the detection of •OH, which was trapped using DMPO, for the different groups. i) The ESR spectra of the detection of ^1^O_2_ in different groups using TEMP (US‐involved group: 1 MHz; 1.2 W cm^−2^; 50% duty cycle; and 1 min).

Taking into consideration the intimate relationship between the enzyme activities and anti‐cancer properties, we explored whether the 2D NiCoO*_x_* nanosheets possess multienzyme‐mimicking activities. The POD‐like activity of NiCoO*_x_* was revealed by the oxidation of tetramethylbenzidine (TMB) upon adding H_2_O_2_ (**Figure** **2**b).^[^ ^25^ ^]^ This oxidant can react with the TMB substrate to generate a blue‐colored product (oxTMB) with a maximum absorption peak at 652 nm. The absorbance at 652 nm in the NiCoO*_x_* + H_2_O_2_ group was significantly higher than in the NiCoO*_x_* or H_2_O_2_ alone groups, validating the dramatic oxidation of TMB (Figure 2c; Figure S2, Supporting Information). Additionally, the POD‐like activity was enhanced by US irradiation (Figure S3, Supporting Information). US can enhance the production of •OH on application of the 2D NiCoO*_x_* nanozymes. Considering the characteristics of the acidic TME, the POD‐mimicking activity of NiCoO_x_ in phosphate buffered saline with different pH values was studied to evaluate the TME adaptability of the engineered nanoenzyme systems. The 2D NiCoO*_x_* nanosheets exhibited only marginal POD‐like activity at neutral pH, while their enzymatic activity was significantly enhanced in weak acidic conditions (Figure 2d). To further investigate the quantity of ROS production under US stimulation and in the presence of NiCoO*_x_*, 1,3‐diphenylisobenzofuran (DPBF) was used to detect the superoxide anion radical (·O_2_^−^) and singlet oxygen (^1^O_2_) after US irradiation (1.2 W cm^−2^, 50% duty cycle, 1 min). The characteristic peak of DPBF at 416 nm at different NiCoO*_x_* concentrations decreased gradually (Figure S4, Supporting Information). Similarly, the absorption of DPBF did not remarkably decline without NiCoO*_x_* (Figure S5, Supporting Information). Subsequently, the US‐augmented OXD‐mimicking activity was also detected for a prolonged time (Figure 2e). 2D NiCoO*_x_* nanosheets possess pH‐responsive catalytic activity, showing a more obvious OXD‐mimicking activity in weak acidic environments (Figures 2b; Figure S6, Supporting Information).

Conversely, CAT is an enzyme that catalyzes the decomposition of H_2_O_2_ to water and O_2_.^[^ ^26^ ^]^ The CAT‐mimicking activity of the 2D NiCoO*_x_* nanosheets was further evaluated by measuring the dissolved oxygen level under the following conditions: 100 µg mL^−1^ of the 2D NiCoO*_x_* nanosheets and 0–100 µm of H_2_O_2_. Similar to the natural catalase, the 2D NiCoO*_x_* nanosheets can efficiently catalyze the conversion of H_2_O_2_ to O_2_, resulting in an increased O_2_ concentration in solution (Figure 2f), confirming the CAT activity of the 2D NiCoO*_x_* nanosheets. Moreover, the generation of O_2_ can relieve tumor hypoxia. GSH, an important antioxidant that is highly expressed in tumor cells, decreased the efficacy of the ROS‐based treatments. We confirmed whether the synthesized 2D NiCoO*_x_* nanosheets have a GSH depletion capability, which could overcome the tumor resistance to oxidation stress and improve the treatment efficacy. Therefore, the GSH depletion capability was quantitatively analyzed using a reduced GSH kit, and the decrease in the NADPH level in the presence of NiCoO*_x_* at 340 nm was measured spectrophotometrically in real time (Figure 2g).^[^ ^27^ ^]^ With an extended time, the characteristic absorbance at 340 nm decreased, indicating that the 2D NiCoO*_x_* nanosheets can undergo a redox reaction associated with GSH depletion (Figure 2g). Moreover, the consumption of GSH increases as the H_2_O_2_ concentration rises, which is reflected in a decreased absorption value (Figure S7, Supporting Information).

Furthermore, the generation of •OH and ^1^O_2_ was quantitatively evaluated based on the electron spin resonance (ESR) measurement (Figure 2h,i), in which •OH was trapped by 5,5‐dimethyl‐1‐pyrroline N‐oxide (DMPO), and 2,2,6,6‐tetra‐methylpiperidine (TEMP) was utilized to capture ^1^O_2_.^[^ ^25^, ^28^ ^]^ As shown in Figure 2h,i, the 2D NiCoO*_x_* nanosheets cannot produce •OH and ^1^O_2_ solely. Unlike other groups, the NiCoO_x_ + H_2_O_2_ or US groups showed strong characteristic signals, indicating the presence of •OH and ^1^O_2_, with the strongest signals being emitted by the NiCoO*_x_* + H_2_O_2_ + US group. Notably, the introduction of US increases the production rate of •OH, as revealed by the enhanced ESR signal strength, which further demonstrates that the external US stimulus (Figures S8 and S9, Supporting Information) combined with the internal cavitation effect can improve the reaction degree and release energy to accelerate the reaction rate, promoting the generation of more ROS and ultimately amplifying the therapeutic efficacy.

These in vitro assays solidly demonstrated the POD‐, OXD‐, CAT‐, and GPx‐like catalytic activities of the 2D NiCoO*_x_* nanosheets (Figure 2a). These activities are further enhanced under weak acidic conditions (Figures S6 and S7, Supporting Information). The ROS family typically contains •OH, ·O_2_^−^, and ^1^O_2_, among which •OH is typically assumed to be the strongest one that can be generated by a Fenton reaction in the presence of H_2_O_2_ and Fenton reagent for US‐mediated cascade catalysis.^[^ ^29^ ^]^ The conversion efficiency of Ni^3+^ to Ni^2+^ and of Co^3+^ to Co^2+^ could be expedited by means of US irradiation, and the produced Ni^2+^ and Co^2+^ further interacts with H_2_O_2_ to make more •OH free radicals available. The use of US in conjunction with powerful shock waves produced from the cavitation bubbles generated by US could trigger partial violent turbulence and increase the mass transfer rate of the nanosystems. Therefore, the engineered 2D NiCoO*_x_* nanosheets with special multienzyme‐mimicking performance could be employed for generating ROS and killing tumor cells, further improving the therapeutic effect induced by US irradiation.

### 2.3 In Vitro Therapeutic Assessment at the Cellular Level

**Figure 3:**
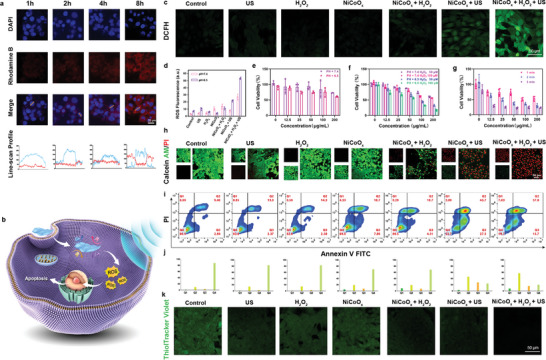
In vitro therapeutic assessment results. a) Representative CLSM images and corresponding linear‐scan profiles of the 4T1 cells after co‐incubation with Rhodamine‐B‐labeled NiCoO*_x_* nanozymes (100 µg mL^−1^) for 1, 2, 4, and 8 h. b) Schematic diagram illustrating the ROS release from the 4T1 cells that causes apoptosis, which is triggered by the US‐enhanced multienzyme activities of the 2D NiCoO*_x_* nanosheets. c) CLSM images of the intracellular ROS level of the 4T1 cells after the different treatments at pH = 6.5 (for the groups involving US: 1 MHz; 1.2 W cm^−2^; 50% duty cycle; 1 min). d) Quantitative ROS production in 4T1 cells treated with the various treatments at pH 6.5 and pH 7.4, respectively. e) Cytotoxicity of the 2D NiCoO*_x_* nanosheets toward 4T1 cells at different concentrations and pHs (6.5 and 7.4) (*n* = 3, mean ± SD). f) Cell viability of the 4T1 cells incubated with different concentrations of 2D NiCoO*_x_* nanosheets at various pHs (6.5 and 7.4) and H_2_O_2_ concentrations (50 and 100 µm) (*n* = 3, mean ± SD). g) Relative cell viabilities of the 4T1 cancer cells after incubation with the 2D NiCoO*_x_* nanosheets at various concentrations and H_2_O_2_ (100 µm) at pH 6.5 using different US irradiation settings (1 MHz; 1.2 W cm^−2^; 50% duty cycle; and 1, 2, and 3 min) (*n* = 4, mean ± SD). h) CLSM images of the 4T1 cancer cells stained with calcein AM/PI at pH 6.5 after the different treatments were performed (for the groups involving US: 1 MHz; 1.2 W cm^−2^; 50% duty cycle; and 1 min) and flow cytometric analysis results of the 4T1 tumor cells after the different treatments at pH 6.5 (for the groups involving US: 1 MHz; 1.2 W cm^−2^; 50% duty cycle; and 1 min). i) The relative quantitative analysis of the 4T1 cancer cells after the various treatments by staining cells with Annexin‐V‐FTIC/PI at pH 6.5. j) Confocal images of the 4T1 tumor cells after co‐incubation with Thiol Tracker violet (green, GSH detection kit) and after the various treatments at pH 6.5 (for the groups involving US: 1 MHz; 1.2 W cm^−2^; 50% duty cycle; and 1 min). The data are shown as the mean ± standard deviation (SD) as calculated using a Student's *t*‐test with *p < 0.05, **p < 0.01, and ***p < 0.001.

Based on its intrinsic non‐toxicity, desirable biodegradability, and water‐solubility, bovine serum albumin (BSA) is the main available albumin extensively used in nanobiomedicine.^[^ ^30^ ^]^ The Fourier transform infrared (FTIR) spectra showed that BSA was successfully grafted onto the surface of the 2D NiCoO*_x_* nanosheets (abbreviated as NiCoO*_x_* @BSA, Figure S10, Supporting Information). The measured zeta potential of NiCoO*_x_* @BSA was around −17.4 mV (Figure S11, Supporting Information). In the cell and animal experiments performed, we used NiCoO*_x_* @BSA because of its improved stability under physiological conditions. The results of the multienzyme‐mimicking activities of the 2D NiCoO*_x_* nanosheets and the synergistic effect enhanced by US were initially evaluated at the cellular level. The cellular uptake of the 2D NiCoO*_x_* nanosheets was first measured. The cellular phagocytosis of this 2D NiCoO*_x_* nanozyme was determined in a breast cancer cell line (mouse 4T1 cells) using confocal laser scanning microscopy (CLSM). As shown in **Figure** **3**a, the Rhodamine B (RB)‐labeled NiCoO*_x_* nanosheets entered the 4T1 cells for each of the different culture durations (1, 2, 4, and 8 h), which can be demonstrated by the red fluorescence emitted by RB and the corresponding linear‐scan spectrogram. The intensity of the red fluorescence gradually increased as the incubation duration increased.

Based on the excellent aforementioned properties of the multienzyme‐activated cytotoxic ROS production, to further investigate the intracellular •OH generation at the cellular level, the ROS levels in cells under acidic (pH 6.5) (Figure 3c) and neutral conditions (pH 7.4) (Figure S12, Supporting Information) were determined based on the fluorescence of a chemical detector, 2′,7′‐dichlorofluorescin diacetate (DCFH‐DA). The single treatments, such as using US, H_2_O_2_, and NiCoO*_x_*, only produce weak fluorescence intensities. Notably, the obvious green fluorescence observed in 4T1 cells indicated that the 2D NiCoO*_x_* nanosheets induce the production of small amounts of ROS under US irradiation, while the further combination with H_2_O_2_ under acidic conditions significantly increases the intracellular ROS levels. Therefore, the chemodynamic effect generated by the 2D NiCoO*_x_* nanosheets and US under acidic conditions (pH 6.5) can markedly improve the generation efficiency of •OH (Figure 3c,d).

The cytocompatibility of 4T1 cells was investigated using a typical cell counting kit‐8 (CCK‐8). As shown in Figure 3e, with the increase in the concentration of the NiCoO*_x_* nanosheets, the viability of 4T1 cells decreased. Next, we investigated the effect of different concentrations of H_2_O_2_ and of the US time on the cytotoxicity. Tumor cells featured higher endogenous H_2_O_2_ levels than normal cells. To further model the TME and more comprehensively understand the cytotoxic properties of the 2D NiCoO*_x_* nanosheets, 4T1 cancer cells were treated using different NiCoO*_x_* concentrations (0, 12.5, 25, 50, 100, and 200 µg mL^−1^) under neutral (pH 7.4) and acidic (pH 6.5) environmental conditions in the presence of H_2_O_2_ (50 or 100 µm) for 24 h (Figure 3f). In an acidic medium, the cytotoxicity assays showed that the cell viability decreased significantly with an increasing sample concentration; however, under neutral conditions, the cell viability did not prominently decline for the same concentration values. By further adding H_2_O_2_, the viability of the 4T1 cancer cells subjected to the 100 µg mL^−1^ NiCoO*_x_* treatment decreased to 44.1% (pH = 6.5, H_2_O_2_ = 50 µm) and 31.9% (pH = 6.5, H_2_O_2_ = 100 µm), respectively (Figure 3f). For the US‐amplified assessment, we further studied the effect of the US time on cell viability (Figure 3g). As the US irradiation time increased, the activity of 4T1 cells decreased significantly, indicating that US could significantly improve the nanoenzyme‐initiated therapeutic efficiency (Figure 3g).

Subsequently, the US‐amplified effects of the 2D NiCoO*_x_* nanosheets on tumor cells were further confirmed using calcein‐AM and propidium iodide (PI) staining experiments (Figure 3h; Figure S13, Supporting Information). Notably, almost all cells of the NiCoO*_x_* + H_2_O_2_ + US group died under acidic conditions due to the increased •OH and ^1^O_2_ production during the US‐amplified catalytic procedure (Figure 3b). The fluorescence results highlight the remarkable US coupling system results. Meanwhile, the apoptosis of 4T1 cancer cells stained with annexin V‐FITC and PI after treatment was measured using flow cytometry (Figure 3i; Figure S14, Supporting Information). A small amount of 4T1 cancer cells died in the 2D NiCoO*_x_* group (Q2 + Q3 quadrant, 26.69%), and the combination of NiCoO*_x_* + H_2_O_2_ and US stimulation effectively amplified the multienzyme‐mimicking activities of the 2D NiCoO*_x_* nanozymes, leading to apoptosis progress revulsion (Q2 + Q3 quadrant, 72.7% for pH = 6.5) (Figure 3j; Figure S15, Supporting Information). Obviously, the US‐induced 2D NiCoO**_x_** treatment regimen had the most prominent tumor cell‐killing capability, killing almost all 4T1 cancer cells, demonstrating that the 2D NiCoO*_x_* nanosheets induced high chemokinetic toxicity and that they could be used as an effective therapeutic agent for the US‐enhanced multienzyme‐catalyzed therapy of malignant tumors.

GSH, which is an important cellular antioxidant, plays an irreplaceable role in protecting cells from intracellular oxidative stress. A superior US‐augmented enzymatic activity was achieved using the 2D NiCoO*_x_* nanosheets. The aforementioned catalysis of the 2D NiCoO*_x_* nanosheets during GSH consumption prompted us to investigate the cellular GSH levels after performing different treatments. The commercial Thiol Tracker Violet intracellular GSH probe was used to visualize the GSH in the 4T1 cells. The control group cells emitted distinct green fluorescence (pH 6.5), whereas the fluorescence intensity observed in the NiCoO_x_ + H_2_O_2_ and NiCoO*_x_* + H_2_O_2_ groups was lower than that found in the control group, and that observed in the NiCoO_x_ + US + H_2_O_2_ group was almost non‐existent, indicating that the 2D NiCoO*_x_* nanosheets have an excellent capability of scavenging the intracellular GSH through their US‐augmented enzymatic activity (Figure 3k; Figure S16, Supporting Information). Therefore, the 2D NiCoO*_x_* nanosheets with multienzyme properties deplete cells of GSH through their US‐augmented enzymatic activity.

### 2.4 Analysis of the In Vitro Pyroptosis‐Like Cancer Cell Death

**Figure 4:**
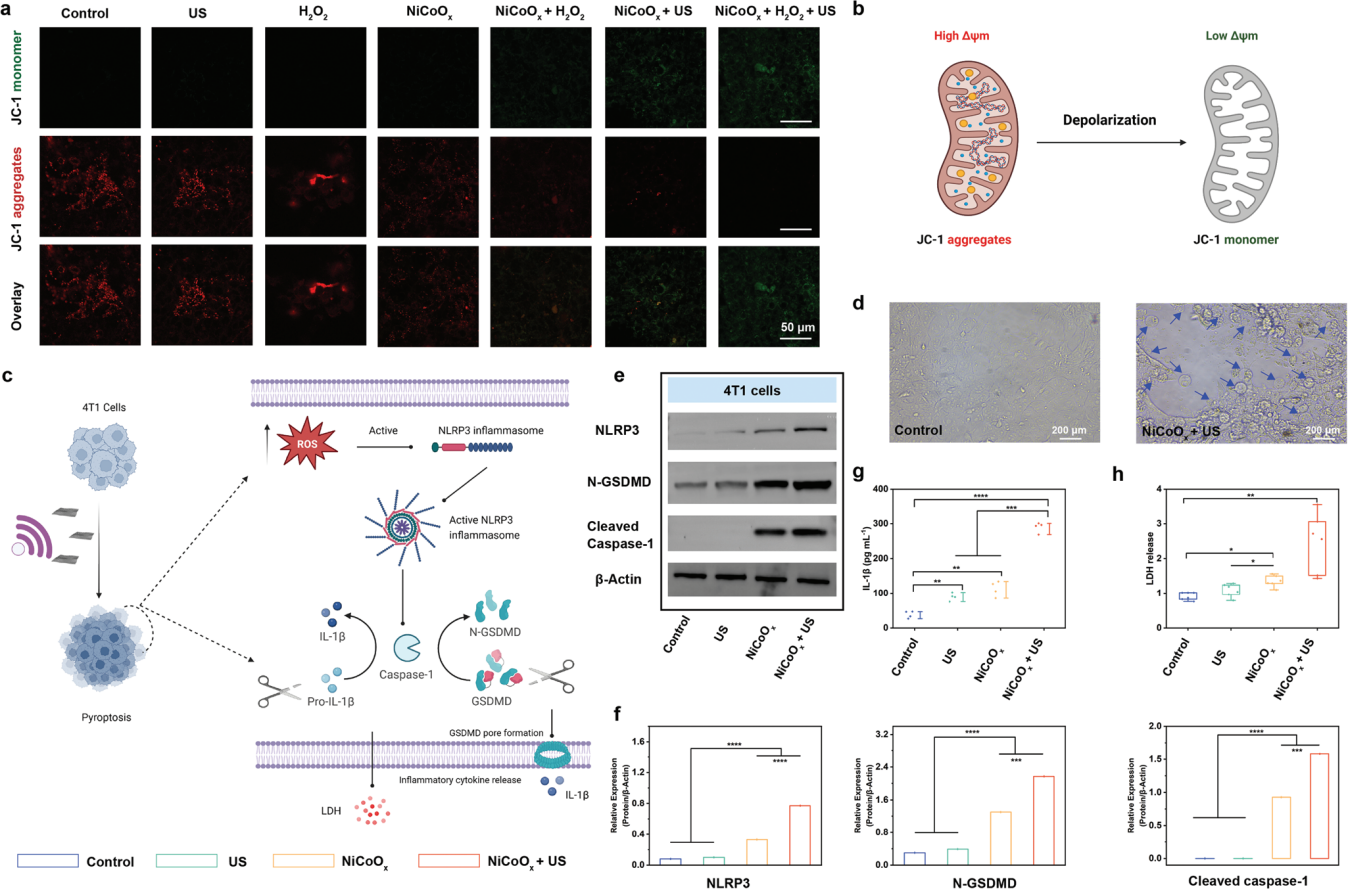
Cellular interaction and the pyroptosis‐inducing performance of the multienzyme‐mimicking 2D NiCoO*_x_* nanosheets. a) JC‐1 assay of the 4T1 tumor cells after coincubation with NiCoO*_x_* (100 µg mL^−1^) via various treatments at pH 6.5 (for the groups involving US: 1 MHz; 1.2 W cm^−2^; 50% duty cycle; and 1 min). b) Diagram of the mitochondrial membrane potential variation. c) Schematic diagram of the molecular mechanism of cancer cell pyroptosis induced by multienzyme‐mimicking NiCoO*_x_* under US irradiation. d) Representative microscopy images (bright‐field) of 4T1 cancer cells after the various treatments. Each arrow tip indicates pyroptotic cells (scale bar: 200 µm). e) The NLRP3, cleaved caspase‐1, and N‐GSDMD protein expression in the various groups of 4T1 tumor cells, as observed in the western blot analysis (for the groups involving US: 1 MHz; 1.2 W cm^−2^; 50% duty cycle; and 1 min). f) Relative contents of NLRP3, cleaved caspase‐1, and N‐GSDMD protein expression. g) Results of the assay analyzing the amount of IL‐1*β* released by the 4T1 tumor cells after the different treatments. h) Results of the assay analyzing the amount of LDH released by the 4T1 tumor cells after the different treatments (for the groups involving US: 1 MHz; 1.2 W cm^−2^; 50% duty cycle; and 1 min). The data are shown as the mean ± standard deviation (SD), as calculated using a Student's *t*‐test with * *p* < 0.05, ** *p* < 0.01, *** *p* < 0.001, and **** *p* < 0.001.

Cell apoptosis is closely related to mitochondrial dysfunction.^[^ ^31^ ^]^ Next, we used JC‐1 staining to detect the mitochondrial membrane potential in the different treatment groups to illustrate the relationship between apoptosis and mitochondrial dysfunction (**Figure** **4**a; Figure S17, Supporting Information). Red JC‐1 aggregates represent healthy high‐potential polarized mitochondria, whereas green JC‐1 monomers represent unhealthy low‐potential depolarized mitochondria (Figure 4b). As shown in Figure 4a, compared with that of the control group, the JC‐1 dye in the NiCoO*_x_* + H_2_O_2_ + US group was completely transformed from red aggregates to green monomers under acidic conditions (pH 6.5), which is representative of mitochondrial membrane disruption. Consequently, the 2D NiCoO*_x_* nanosheets were significantly cytotoxic in acidic medium, whereas they did not lead to significant cell death in neutral medium (Figure S17, Supporting Information). Quantitative fluorescence analysis further confirmed this (Figure S18, Supporting Information). These in vitro results suggest that the US‐induced 2D NiCoO*_x_* nanosystems can achieve GSH depletion and amplify the oxidative stress, thereby promoting US‐augmented cancer cell killing. The 2D NiCoO*_x_* nanosheets cause obvious mitochondrial damage, which could be attributed to ROS production. Additional significant upstream processes connected to NLRP3 activation include mitochondrial malfunction and the release of ROS into the cytosol.^[^ ^32^ ^]^ Considering the desirable US‐amplified performance of the 2D NiCoO*_x_* nanosheets, these are speculated to be able to induce pyroptosis. The formation of transmembrane pores is the main feature of pyrolysis, which leads to the swelling and expansion of cells and to bubble‐like protrusions.^[^ ^33^ ^]^ To verify our hypothesis, we used an inverted microscope to examine the morphology of the 4T1 cells of the different treatment groups. Compared with the results of the control group, a larger number of vesicles and distinct balloon‐like cells were observed in the NiCoO*_x_* + US group, which demonstrates that the NiCoO*_x_* + US treatment could efficiently induce pyroptosis (Figure 4d).

GSDMs are a family of intracellular proteins with a pore‐forming effect that can induce cell membrane permeability and pyroptosis. GSDMs have a poisonousness N‐terminal structure (N‐GSDMD) and a C‐terminal repressor structure (C‐GSDMD), which can be proteolytically activated by certain proteases.^[^ ^34^ ^]^ The released N‐GSDMD forms large oligomeric pores in the plasma membrane, rupturing the cell membrane. The cells undergoing pyrolysis release various pro‐inflammatory cytokines into the extracellular environment, thus triggering inflammation and an immune response (Figure 4c). To further understand the 2D NiCoO*_x_*‐mediated pyroptosis mechanism, western blot analysis of the related pyrolytic core proteins was performed (Figure 4e). The expression of pyrolysis biomarkers, including NLRP3 and N‐GSDMD, was significantly increased in the 4T1 cells of the NiCoO*_x_* group. Under US irradiation (NiCoO*_x_* + US), their pyroptosis capability could be further improved (Figure 4f; Figure S19, Supporting Information). Furthermore, we also observed that the expression of cleaved caspase‐1 in the NiCoO*_x_* + US group was enhanced. Caspase‐1 could cleave GSDMD, and the formed pores led to cell pyroptosis and released DAMPs.^[^ ^35^ ^]^ Another characteristic of GSDMD‐dependent pyroptosis is that it promotes the non‐conventional secretion of inflammatory molecules and cell contents, thus causing intense inflammation.^[^ ^36^ ^]^ After the NiCoO*_x_* + US treatment, the concentrations of lactic dehydrogenase (LDH) and interleukin‐1*β* (IL‐1*β*) in the 4T1 cells apparently increased; however, no significant release of LDH or IL‐1*β* was observed in the control group, indicating that the NiCoO*_x_* + US treatment highly induces pyroptosis (Figure 4g,h). All of these results confirm that the NiCoO*_x_* + US treatment can lead to pyroptosis.

### 2.5 RNA Sequencing and Analysis of the Therapeutic Mechanism

**Figure 5:**
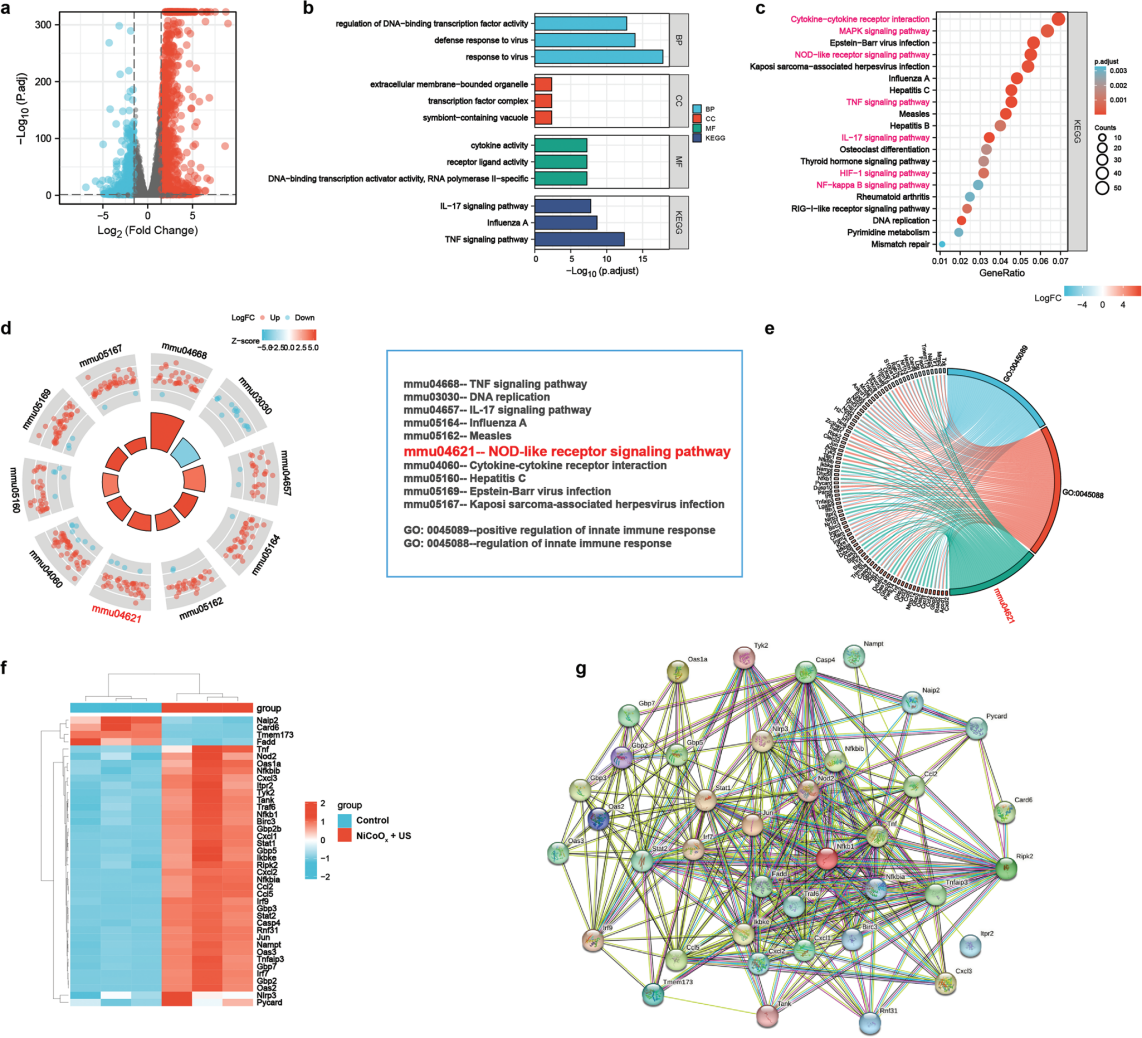
Exploration of the therapeutic mechanism of the US‐induced 2D NiCoO*_x_* nanosheets on the 4T1 cells via transcriptome high‐throughput sequencing. a) The volcano plot of the up‐ and down‐regulated genes in the US‐induced NiCoO*_x_* group (1 MHz, 1.2 W cm^−2^, 50% duty cycle, and 1 min) and comparison with that of the control group. b) Gene ontology (GO) and Kyoto Encyclopedia of Genes and Genomes (KEGG) analyses of the differentially expressed genes in the US‐induced NiCoO*_x_* group and control group (BP, biological process; CC, cellular components; MF, molecular function). c) KEGG pathway enrichment analysis of the identified differentially expressed genes in the US‐induced NiCoO*_x_* group and control group. d) The circle plot of GO and KEGG analysis. e) Enriched chord plot of the KEGG pathways. f) The heat map of the differentially expressed genes. g) Protein–protein interaction network of the 40 functional intersecting genes.

To further clarify the underlying cellular therapeutic mechanism of US‐induced NiCoO_x_ multienzyme‐mimicking nanosystems, transcriptome analysis was performed to compare the changes in the messenger RNA (mRNA) expression in 4T1 cancer cells treated with 2D NiCoO*_x_* in the presence of H_2_O_2_. In total, 1786 genes were differentially expressed from those expressed in the US‐treated NiCoO*_x_* group, including 1104 up‐regulated mRNAs and 682 down‐regulated mRNAs (**Figure** **5**a; Table S1, Supporting Information). Based on the diversity of the RNA sequencing results, we performed Gene Ontology (GO) and Kyoto Encyclopedia of Genes and Genomes (KEGG) analyses to explain the changes in the mRNA biological function and the relevant pathways of influence (Figure 5b; Table S2, Supporting Information). The information acquired from the aforementioned analysis results (Figure 5c) suggests that the differentially expressed genes observed after combining the NiCoO_x_ with the US treatment were enriched in inflammatory signaling pathways, such as cytokine‐cytokine receptor interaction, MAPK signaling pathway, NOD‐like receptor signaling pathway, TNF signaling pathway, IL‐17 signaling pathway, and NF‐kappa B signaling pathway, which verifies that the NiCoO*_x_* + US treatment efficiently kills cancer cells and induces inflammation through 2D NiCoO*_x_*‐induced pyroptosis. Interestingly, the HIF‐1 signaling pathway was also enriched for those genes, indicating that the oxygen level of the tumor tissues could change after the treatment (Figure 5c). The up‐ and down‐regulated pathway‐related genes are more simply reported in the loop diagram (Figure 5d). Notably, the results affirm that the association of NiCoO*_x_* with US aggravates inflammation, which is reflected in the upregulation of the NOD‐like receptor signaling pathway, confirming the superimposed effect of the combined application of NiCoO*_x_* and US.

After determining the gene expression differences, the specific regulatory genes corresponding to the main influencing pathways were presented in a chord diagram after the KEGG pathway analysis. Notably, the most significantly regulated genes belonged to the NOD‐like receptor signaling pathway (Figure 5e; Table S3, Supporting Information). The gene set enrichment analysis results also confirmed that the significantly differentially expressed genes were involved in the NOD‐like signaling pathway (Figure S20, Supporting Information). To further explore the underlying mechanism, a heatmap and protein–protein interaction system of the differentially expressed genes related to PCD (including inflammatory response and pyroptosis) were generated (Figure 5f,g). Notably, NLRP3 was remarkably up‐regulated, which is consistent with the western blot results (Figure 4e,f). For the nuclear factor‐k‐gene binding (NF‐kB)‐related genes (*Nfkbib*, *Nfkb1*, *Ripk2*, and *Nfkbia*), tumor necrosis factor (TNF)‐related genes (*Tnf*, *Traf6*, and *Tnfaip3*), the antiviral protein genes (*Oas1a*, *Oas3*, and *Oas2*), interferon‐related genes (*Tyk2*, *Gbp2b*, *Stat1*, *Gbp5*, *Irf9*, *Stat2*, *Gbp7*, *Irf7*, and *Gbp2*), and chemokine genes (*Cxcl3*, *Cxcl1*, *Cxcl2*, *Ccl2*, and *Ccl5*), the obvious appearance of these up‐regulated gene changes confirmed that the 2D NiCoO*_x_* nanosheets can activate a pyroptosis‐binding inflammatory reaction. These findings demonstrate that the US‐induced 2D NiCoO*_x_* nanosheets can alleviate tumor hypoxia and further induce pyroptosis.

### 2.6 In Vivo Antitumor Effect

**Figure 6:**
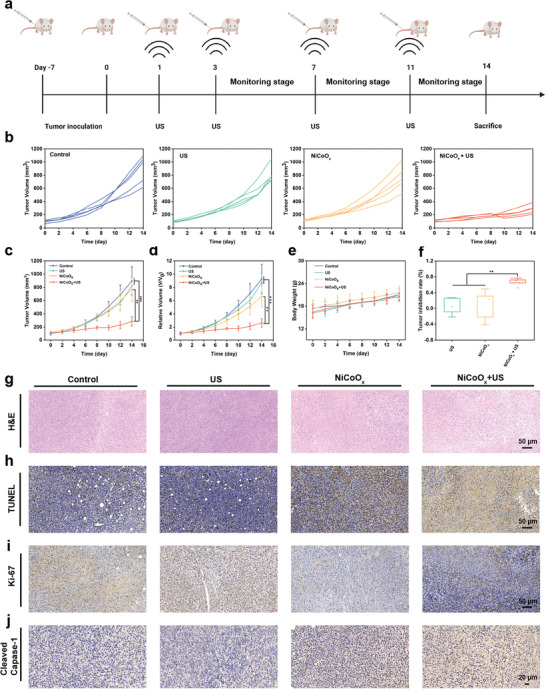
Evaluation of the in vivo antitumor efficacy. a) Schematic diagram of the timeline of the assay performed to evaluate the effectiveness of 2D NiCoO*_x_* nanoenzymes for antitumor therapy. b) Individual tumor growth curve of the tumor‐bearing mice in the various treatment groups after being exposed to the US‐amplified multienzyme effect for tumor treatment (*n* = 5). c) Tumor volume and d) relative tumor volume of the mice after undergoing diverse treatments (*n* = 5). e) Body weight of mice during the 14 days of treatment. f) Relative 4T1‐tumor inhibition rate of the treatment group and comparison with that of the control group. g) H&E, h) TUNEL, i) Ki‐67, and j) cleaved caspase‐1 staining images of the tumor slices obtained from the various groups (control, US, NiCoO*_x_*, and NiCoO*_x_* + US) of mice.

Considering the favorable catalytic and cellular therapeutic results obtained using the 2D NiCoO*_x_* nanosheets, we further evaluated their antitumor therapeutic effect in vivo. Female BALB/c mice aged 3–4 weeks were subcutaneously injected with 4T1 cells on the right side to establish a breast cancer model, and the tumor‐bearing mice were irregularly divided into four groups (*n* = 5 per group): 1) control group; 2) US group; 3) NiCoO*_x_* group; 4) NiCoO*_x_* + US group (intravenous injection (i.v.)). The detailed treatment protocol of the tumor‐bearing mice is shown in **Figure** **6**a. After intravenous injection of NiCoO*_x_* nanosheets, the majority of Ni was distributed in the spleen and liver tissues of the 4T1 tumor‐bearing mice, in addition to their efficient accumulation at tumor sites (Figure S21, Supporting Information). The tumor was irradiated with assisted US irradiation (1 MHz, 1.2 W cm^−2^, 50% duty cycle, and 10 min) 1, 3, 7, and 11 days after tail vein injection of a NiCoO*_x_* aqueous solution for 15 min and 4 h. The body weight and tumor volume of the mice were monitored every 2 days during the whole treatment period. As expected, no apparent body weight changes were observed for any of the groups, which show the high therapeutic biosafety of the treatment (Figure 6d). Concurrently, compared with the control group results, the results of the hematoxylin and eosin (H&E) staining of the main organs (heart, liver, lungs, spleen, and kidneys) of the mice further showed no obvious pathological abnormalities or inflammatory lesions in the treatment group, confirming the desirable histocompatibility of the 2D NiCoO_x_ nanosheets (Figure S22, Supporting Information).

Regarding tumor inhibition evaluation, the tumor growth of each group during the 14 days treatment period was recorded in detail (Figure 6b–e). Compared with the control group mice, for which a continuous growth of the tumor volume was observed, the mice administered NiCoO*_x_* showed slight tumor inhibition (average volume of 288 mm^3^), which is attributed to the catalytic therapeutic effect of the 2D NiCoO*_x_* nanozymes (Figure 6b). A more effective 4T1 breast tumor inhibition was observed in the group treated with NiCoO*_x_* + US, especially when NiCoO*_x_* was administered intravenously. After 14 days of treatment, the average tumor volume of mice decreased to 107 mm^3^ (Figure 6c), highlighting how US magnifies the simulated activity of the 2D NiCoO_x_ nanozymes with POD‐, OXD‐, GPx‐, and CAT‐mimicking activities. This satisfactory tumor inhibition effect is attributed to the synergistic effect of US‐enhanced nanocatalytic therapy, which is enabled by the 2D NiCoO*_x_* nanozymes. Furthermore, we calculated the tumor growth inhibition (TGI) rate to quantitatively evaluate the desirable antitumor therapeutic efficiency of the nanoenzymes enhanced by US. Compared with that of the NiCoO*_x_* group (the relative TGI rate was 0.14%) at the end of day 14, the TGI rate of the US‐induced NiCoO*_x_* group was 71.8%, surpassing that of the US group (−0.04%) (Figure 6f). Moreover, the average weight of the tumors collected after treatment based on relevant visualized tumor photographs further confirmed the high antitumor efficacy (Figure S23, Supporting Information).

Additionally, the results of H&E and TdT‐mediated dUTP nick‐end labeling (TUNEL) staining of the tumor slices after treatment further suggest that the tumors in the NiCoO*_x_* + US group show serious apoptosis (Figure 6g,h; Figure S24, Supporting Information). Furthermore, the ROS level in NiCoO*_x_* + US‐treated tumors is considerably higher than that in NiCoO*_x_* and US‐treated tumors, deriving from the in vivo O_2_^−^ overproduction triggered by NiCoO*_x_* + US (Figure S25 Supporting Information). And the tumor cell proliferation inhibition effect was confirmed by the Ki‐67 immunohistochemistry staining assay results. As expected, compared with those of the control and US groups, an effective inhibitory effect on tumor proliferation, characterized by a strong blue signal, was observed in the mice injected with the 2D NiCoO*_x_* nanozymes under US stimulation (Figure 6i; Figure S26, Supporting Information). To further confirm the pyroptosis effect, we also carried out immunohistochemical examination of cleaved caspase‐1 expression in the tumor tissue (Figure 6j; Figure S27, Supporting Information). The expression of this protein was increased in the NiCoO_x_ group, and its effect could be further drastically enhanced under US irradiation, demonstrating that the NiCoO*_x_* + US treatment can effectively induce cancer cell pyroptosis. Taken together, we can conclude that the NiCoO_x_ + US‐mediated pyroptosis could effectively induce a lasting antitumor effect.

## 3 Conclusions

In summary, we developed specific US‐augmented multienzyme‐mimicking strategies for an efficient tumor nanotherapy by engineering distinct 2D NiCoO nanosheets/nanoenzymes to improve the rate of harmful ROS generation for catalytic tumor nanotherapy and cell pyroptosis. A variety of multienzyme‐mimicking activities were observed in the designed/fabricated 2D NiCoO*_x_* nanosheets, including ROS‐associated POD‐ and OXD‐mimicking activities, hypoxia reversal involving a CAT‐like activity, and a GSH‐consumption capability involving GPx‐like activity. US stimulation improved the multienzyme‐like activities and enhanced ROS production, which caused mitochondrial dysfunction. The pyroptosis process can be specifically induced by the ROS generated by the multienzyme‐mimicking with US activation, which was demonstrated by caspase‐1 activation and the release of N‐GSDMD and LDH/IL‐1*β*. The subsequent transcriptome sequencing analysis revealed that pyroptosis was caused by the ROS‐NLRP3‐GSDMD pathway. Importantly, the US‐augmented catalytic tumor nanotherapy produced remarkable therapeutic results, as evidenced by the systematic in vitro and in vivo evaluations (4T1 breast tumor model). Collectively, our results show that the engineered 2D nanosheets/nanoenzymes with US‐augmented multienzyme‐mimicking activities constitute a novel strategy to induce pyroptosis, which enables a highly efficient catalytic tumor nanotherapy.

## Conflict of Interest

The authors declare no conflict of interest.

## Supporting information

Supporting Information

## Data Availability

The data that support the findings of this study are available from the corresponding author upon reasonable request.
