# Supplementary material for: Engineering 2D Multienzyme‐Mimicking Pyroptosis Inducers for Ultrasound‐Augmented Catalytic Tumor Nanotherapy
Source: Adv Sci (Weinh). 2023 Jun 23;10(24):2301279. doi: 10.1002/advs.202301279 (PMC10460896; doi:10.1002/advs.202301279)
Supplement: Supplementary file 1 — Supporting Information [file ADVS-10-2301279-s001.pdf]

ADVANCED SCIENCE
Open Access

Supporting Information

for *Adv. Sci.*, DOI 10.1002/advs.202301279

Engineering 2D Multienzyme-Mimicking Pyroptosis Inducers for Ultrasound-Augmented Catalytic Tumor Nanotherapy

Xinran Song, Hui Huang, Lili Xia, Wencong Jia, Shaoling Yang, Chenglong Wang* and Yu Chen**

Supporting Information

Engineering 2D Multienzyme-mimicking Pyroptosis Inducers for Ultrasound-augmented Catalytic Tumor Nanotherapy

Xinran Song, Hui Huang, Lili Xia, Wencong Jia, Shaoling Yang, Chenglong Wang* and Yu Chen**

X. Song, H. Huang, L. Xia, W. Jia, and Prof. Y. Chen
School of Environmental and Chemical Engineering, Shanghai University, Shanghai 200444, P. R. China. Email: chenyuedu@shu.edu.cn (Y. Chen).

A/Prof. C. Wang
Department of Orthopedic Surgery, XinHua Hospital Affiliated with Shanghai Jiaotong University School of Medicine, Shanghai 200082, P. R. China. Email: wangchenglong@xinhuamed.com.cn (C. Wang).

Prof. S. Yang
Department of Ultrasound Medicine, Shanghai Eighth People's Hospital, Shanghai 200235, P. R. China. Email: drysl@163.com (S. Yang).

Experimental section

Materials. All required reagents were purchased from commercial sources without further purification. Cobaltous nitrate hexahydrate ($Co(NO_3)_2{\cdot}6H_2O$), nickel nitrate hexahydrate ($Ni(NO_3)_2{\cdot}6H_2O$), absolute ethanol, urea (CH_4N_2O), poly(vinylpyrrolidone) (PVP, Mw=55000), 3,3′,5,5′-tetramethyl-benzidine (TMB), Rhodamine B (RhB), 2,7-dichlorofluorescin diacetate (DCFH-DA), 1, 3-diphenylisobenzofuran (DPBF) and bovine serum albumin (BSA) were purchased from Sigma-Aldrich. Fetal bovine serum (FBS) and high glucose dulbecco's modified eagle's medium (DMEM) were purchased from Gibco Life Technologies. The commercial enzyme-linked immunosorbent assay (ELISA) kit of interleukin-1β (IL-1β), Calcein-AM and propidium iodide (PI) kit, 4',6-diamidino-2-phenylindole (DAPI), 2,7-dichlorofluorescein diacetate (DCFH-DA), bicinchoninic acid (BCA) protein assay kit, lactic dehydrogenase (LDH) cytotoxicity assay kit, glutathione peroxidase (GPx) assay kit and JC-1 staining kit, Cell Counting Kit-8 (CCK-8), mouse breast cancer cells (4T1) were purchased from Beyotime Biotechnology (Shanghai, China). Thiol TrackerTM Violet and annexin V-FITC/PI apoptosis detection kit were obtained from Thermo Fisher Scientific (USA).

Synthesis of $NiCoO_x$ nanosheets. First, 300 mg $Ni(NO_3)_2{\cdot}6H_2O$ and 600 mg $Co(NO_3)_2{\cdot}6H_2O$ were dissolved in 10 ml ultra-pure water and 70 ml absolute ethanol. After stirring for a while, 1 g of urea and 1 g of PVP were added into the solution. The mixture was added to a round-bottom flask, stirred at 90 □, and refluxed for 10 h. Then, the resulting product was centrifuged and followed by being washed with absolute ethyl alcohol and DI water. At last, the product was freeze and calcined at 300 □ for 3 h with a heating rate of 2 □$\cdot$$min^{-1}$. To conjugate bovine serum albumin (BSA), the $NiCoO_x$ solution was mixed with BSA aqueous solution at the weight ratio of 1/1, and the reaction was stirred at room temperature for 24 hours. The mixture was centrifuged under 10000 rpm for 15 min, and the precipitation was washed three times with DI water and ethanol separately. After that, the BSA-modified $NiCoO_x$ ($NiCoO_x$@BSA) was obtained after lyophilization.

Characterization. Transmission electron microscope (TEM) images showing the material morphology and structure were acquired by the Tecnai G220 (Shimadzu, Japan). The XPS was

taken from an ESCALAB 250 Xi Mg (Thermo Scientific, Japan) to determine the chemical composition. And the X-ray diffraction (XRD) was tested via a Rigaku D/MAX-2550 V X-ray power diffractometer. Fourier transform infrared spectroscopy (FTIR) of nanoparticles was recorded by the FTIR-8300 spectrometer (Bruker). The ultraviolet-visible (UV-vis) spectrometer was used to record UV-vis absorption spectra. Dynamic light scattering (DLS) and zeta potential were obtained by the Zetasizer Nano-ZS90 (Malvern). Meanwhile, the electron spin resonance (ESR) spectrum was acquired and measured on a Bruker EMX1598 spectrometer. The fluorescence images were conducted by a confocal scanning microscope (Leica TCS SP8). The western blotting was performed via AI600 (GE, America).

Multienzyme-mimicking ability.

Catalase (CAT)-mimicking activity of 2D $NiCoO_x$ nanosheets. The CAT-mimicking activity of 2D $NiCoO_x$ nanosheets was dynamically measured in PBS buffer containing 100 μg mL^{-1} $NiCoO_x$ and different concentrations of H_2O_2 (0, 25, 50, and 100 μM) to prove $NiCoO_x$-catalyzed O_2 production. The portable dissolved oxygen meter (JPB-607A) was used to dynamically measure the level of dissolved oxygen produced by the decomposition of H_2O_2.

Peroxidase (POD)-mimicking activity of 2D $NiCoO_x$ nanosheets. The POD-mimicking activity of 2D $NiCoO_x$ nanosheets was measured by UV-vis absorption spectra to obtain the absorbance of 3,3′,5,5′-tetramethylbenzidine (TMB) solution. H_2O_2 solution (10 mM) with $NiCoO_x$ nanosheets (14 mg mL^{-1}, 30 μL) and TMB (8 mM) was added into the colorimetric dish and then the absorbance at 652 nm was measured from a UV/vis scanning spectrophotometer. The US parameters are 1.2 W cm^{-2}, 50% duty cycle, 1 min.

Oxidase (OXD)-like activity of 2D $NiCoO_x$ nanosheets. The OXD-mimicking activity of 2D $NiCoO_x$ nanosheets was measured by UV-vis absorption spectra to obtain the absorbance of DPBF solution at 420 nm. In addition, the OXD-like activity of $NiCoO_x$ nanosheets was measured by UV-vis absorption spectra to obtain the absorbance of 3,3′,5,5′-tetramethylbenzidine (TMB) solution. PBS with different pH values (4.4, 5.4, 6, 6.5, and 7.4)

and $NiCoO_x$ nanosheets (2 mg mL^{-1}, 30 μL) and TMB (8 mM) were added into the colorimetric dish and then the absorbance at 652 nm was measured from a UV/vis scanning spectrophotometer.

Glutathione peroxidase (GPx)-mimicking of 2D $NiCoO_x$ nanosheets. The GPx-like ability was measured by using the GPx assay kit, and the reactant was spectrophotometrically monitored at 340 nm using UV-vis spectroscopy.

ROS generation capacity of 2D $NiCoO_x$. The experiment was divided into five groups: (1) control, (2) US (1 MHz; 1.2 W cm^{-2}; 50% duty cycle; 1 min), (3) H_2O_2 (100 μM), (4) $NiCoO_x$, (5) $NiCoO_x$ + H_2O_2 (100 μM), (6) $NiCoO_x$ + US (1 MHz; 1.2 W cm^{-2}; 50% duty cycle; 1 min) and (6) $NiCoO_x$ + US (1 MHz; 1.2 W cm^{-2}; 50% duty cycle; 1 min) + H_2O_2 (100 μM). The concentration of $NiCoO_x$ was set as 100 μg mL^{-1}, and US irradiation was 1.2 W cm^{-2}, 50% duty cycle, 1 min. For the measurements of ·OH and $^1O_2$, 2,2,6,6-tetramethylpiperidine (TEMP) or 5,5-dimethylpyrroline N-oxide (DMPO) were used, and electron spin resonance (ESR) was performed after the addition of TEMP or DMPO by using an ESR spectrometer (Bruker EMX1598).

Cell culture, cell uptake, cytotoxicity assay, and apoptosis assay of 2D $NiCoO_x$. Mouse breast carcinoma cells (4T1 cells) were cultured with high glucose dulbecco's modified eagle's medium (DMEM) supplemented with 10% fetal bovine serum (FBS), 100 U mL^{-1} streptomycin, and 100 mg mL^{-1} spenicillin in 5% CO_2 atmosphere at 37 °C. Then the cell culture medium was replaced with fresh medium every 2 days. In order to assess the cellular internalization process of $NiCoO_x$, the surface of the $NiCoO_x$ was embellished with Rhodamine B (RB). Appropriate amounts of RB and $NiCoO_x$ were treated in ethanol solution, and the $NiCoO_x$-RB conjugate was obtained after magnetic stirring for 12 h in the dark condition. 4T1 cells were planted in a confocal dish overnight, and RB-labeled $NiCoO_x$ (100 μg mL^{-1}) was incubated with the cells (0, 1, 4, and 8 h). After incubation, tumor cells were stained with 4',6-diamino-2-phenylindole (DAPI) for 15 min, washed twice with PBS, and fixed with glutaraldehyde. Finally, the cells

were observed by CLSM. For cytotoxicity assay, 4T1 cells were seeded into 96-well plates at a density of 1 × 10^4 cells per well and individually incubated at 37 °C in 5% CO_2. Cells were incubated with different concentrations of $NiCoO_x$ (0, 12.5, 25, 50, 100, and 200 ug mL^{-1}), different medium environments (acidic conditions and neutral conditions of 50 or 100 μM H_2O_2), and different ultrasound irradiation durations (1, 2, and 3 min) for 24 h. Then, cytotoxicity was measured by the cell counting kit-8 (CCK-8) method. To verify $NiCoO_x$-induced apoptosis, 4T1 cells (1 × 10^6) were incubated with $NiCoO_x$ (100 μg mL^{-1}) in different groups for 14 h, then washed twice with PBS and immediately dyed with Annexin V-FITC/PI kit, and then detected using a flow cytometer (BD Accuri C6).

Detection of intracellular ROS. 4T1 cells were seeded into confocal dish and cultured with H_2O_2 and $NiCoO_x$ (100 μg mL^{-1}) incubated in 5% CO_2 at 37 °C, pH of 7.4 and 6.5 for 12 h, respectively. Cells are processed differently including PBS, $NiCoO_x$, H_2O_2 (100 μM), US (1 MHz; 1.2 W cm^{-2}; 50% duty cycle; 1 min), $NiCoO_x$ + H_2O_2 (100 μM), $NiCoO_x$ + US (1 MHz; 1.2 W cm^{-2}; 50% duty cycle; 1 min), and $NiCoO_x$ + H_2O_2 (100 μM) + US (1 MHz; 1.2 W cm^{-2}; 50% duty cycle; 1 min). Subsequently, the cells were washed with PBS and incubated with DCFH-DA (10 μM) at 37°C for 20 min. Then, the fluorescence intensity of cells was assessed via detection of the fluorescence signal intensity of DCF via CLSM.

JC-1 staining. 4T1 cells were seeded in confocal dishes (Wuxi NEST Biotechnology Co., Ltd.), after 12 hours of incubation, DMEM was then replaced with new $NiCoO_x$-containing fresh DMEM (pH6.5 or pH7.4). After 12 h, cells incubated with JC-1 dye at 37°C for 20 min and then cells were washed with PBS and observed using a CLSM.

GSH depletion capacity of 2D $NiCoO_x$. The experiment was divided into five groups under acidic conditions (pH 6.5): (1) control, (2) US (1 MHz; 1.2 W cm^{-2}; 50% duty cycle; 1 min), (3) H_2O_2 (100 μM), (4) $NiCoO_x$, (5) $NiCoO_x$ + H_2O_2 (100 μM), (6) $NiCoO_x$ + US (1 MHz; 1.2 W cm^{-2}; 50% duty cycle; 1 min) and (6) $NiCoO_x$ + US (1 MHz; 1.2 W cm^{-2}; 50% duty cycle; 1 min) + H_2O_2 (100 μM). The concentration of $NiCoO_x$ was set as 100 μg mL^{-1}, and US irradiation

was 1.2 W cm^{-2}, 50% duty cycle, 1 min. Then labeling cells with 20 μM ThiolTracker™ Violet dye for 30 minutes at 37˚C. The ThiolTracker™ Violet dye working solution was replaced with a suitable buffer or medium. Last, the cells are now ready for imaging using a CLSM.

Gene sequencing. 4T1 cells were seeded into the 6-well plates (1 × 10^5 cells per well) and incubated for 24 h. Next, after different treatments (control, $NiCoO_x$ + US), the cells were washed with PBS, and the cells were collected according to the requirements of sample delivery for gene sequencing (Personalbio Biotechnology Co., Ltd.). The US parameters are 1.2 W cm^{-2}, 50% duty cycle, 1 min.

Western blot analysis. 4T1 cells were seeded into the 6-well culture plate. After various treatments (control, US, $NiCoO_x$, $NiCoO_x$ + US), the cells were collected and resuspended in RIPA buffer containing protease inhibitors, and lysed at 4 □ for 30 minutes, then centrifuged (4 □, 13000r, 10 min) to remove debris. The equivalent amount of protein cell extract was diluted in the sample buffer and Western Blot assay was carried out in accordance with the standard procedure. The antibodies used were anti-cleaved N-terminal GSDMD antibody (Abcam, [EPR20829], ab215203), anti-NLRP3 antibody (Abcam, [EPR23073], ab270449), and anti-cleaved caspase-1 (Cell Signaling Technology, Asp297). The Image J software was used to quantify the density measurement value of the obtained bands. The US parameters are 1.2 W cm^{-2}, 50% duty cycle, 1 min.

Cytokines release and lactic dehydrogenase (LDH) assays. 1×10^4 4T1 cells were inoculated into each well of 96 well plate. After 24 h of seeding, the supernatant of cell culture was collected after different treatments. The amount of IL-1β in culture supernatants was determined by ELISA kit and LDH release was detected by LDH cytotoxicity assay kit to verify cell pyroptosis. The US parameters are 1.2 W cm^{-2}, 50% duty cycle, 1 min.

Anti-tumor effect. BALB/c mice (female, 3-4 weeks) were purchased from Shanghai Jiesijie Laboratory Animal Co., Ltd. 1 × 10^6 4T1 cells were injected subcutaneously per mouse. When tumors reached about 60-70 mm^3, the mice were divided into five groups (n = 5 per group)

randomly: 1) control group; 2) US group; 3) $NiCoO_x$ group; 4) $NiCoO_x$ + US group (intravenous injection, i.v.). Healthy female Balb/c mice were intravenously injected with $NiCoO_x$@BSA nanocomposites (10 mg/kg, 100 μL). The body weight and tumor volume of mice were monitored every 2 days during the whole treatment period. The tumor volume was calculated by $L \times W^2 \times 0.5$ (L: the length of the tumor; W: the width of the tumor), respectively. On day 14, mice in each group were euthanized, tumors and main organs were taken out, then record tumor weight and take photos. Finally, the tumor sections were stained with H&E, TUNEL and Ki-67 staining. The US parameters are 1.2 W cm^{-2}, 50% duty cycle, 10 min.

Statistical analysis. The mean value and standard deviation (mean ± SD) of all data showed that the statistical analysis * $p<0.05$ of different groups through Graphpad Prism 9. was considered statistically significant, * * $p<0.01$ and * * * $p<0.001$, * * * * $p<0.001$ were considered notably significant.

Supplementary figures

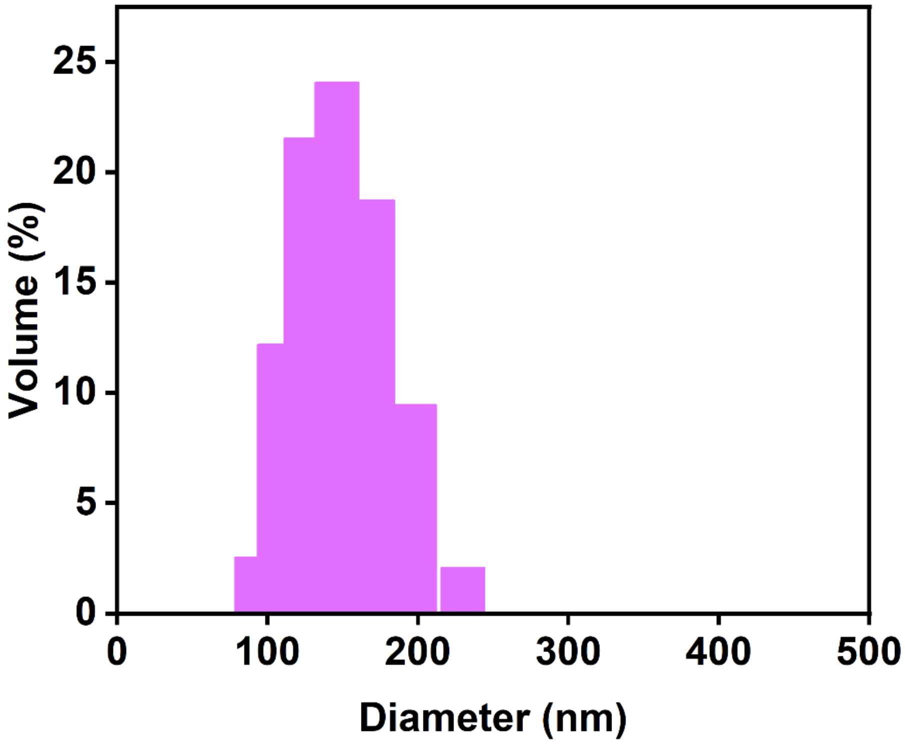

Figure S1. Size distribution of 2D $NiCoO_x$.

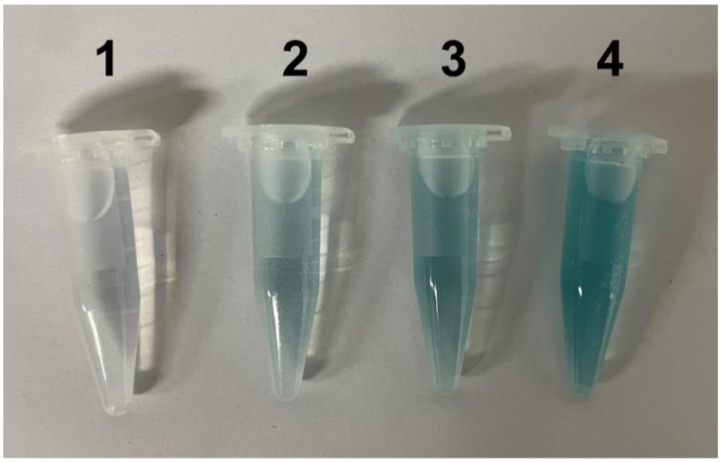

Figure S2. The corresponding digital photos of POD-mimicking activity of 2D $NiCoO_x$ nanosheets tested by TMB probe (1: TMB + H_2O_2, 2: TMB + $NiCoO_x$, 3: TMB + H_2O_2 + $NiCoO_x$, 4: TMB + H_2O_2 + $NiCoO_x$ + US).

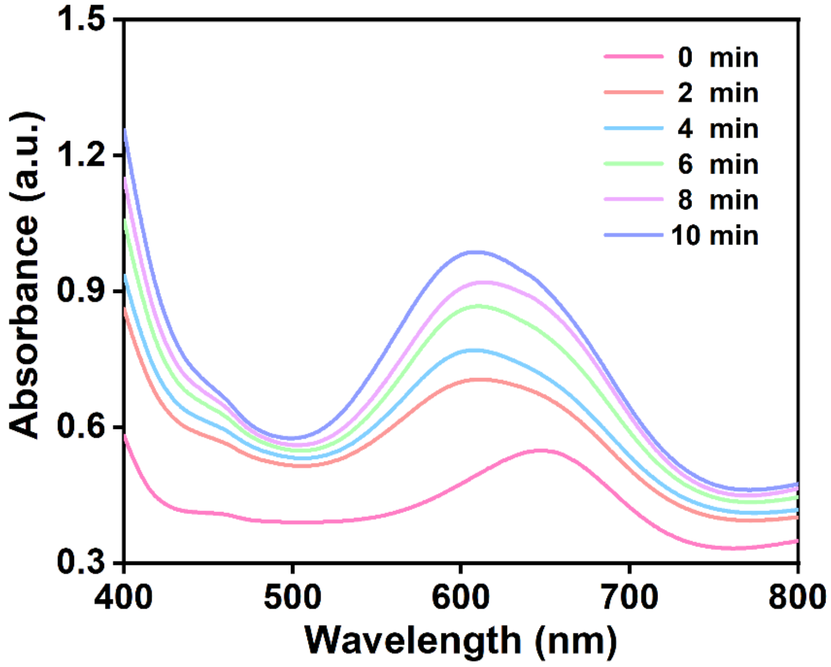

Figure S3. Time-dependent absorbance changes at 652 nm by TMB probe with 2D $NiCoO_x$ under US activation.

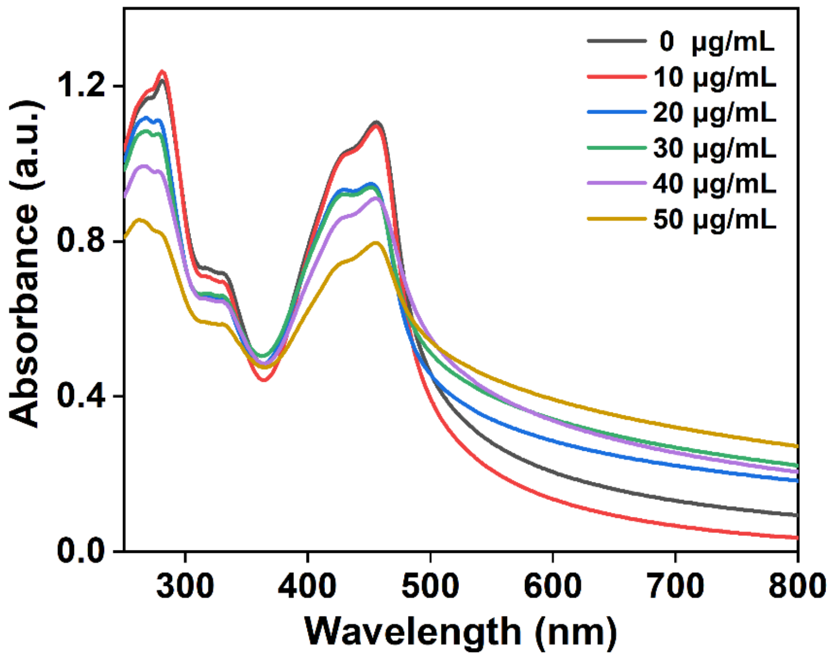

Figure S4. Absorption attenuation of fluorescent probe DPBF at different 2D $NiCoO_x$ concentrations.

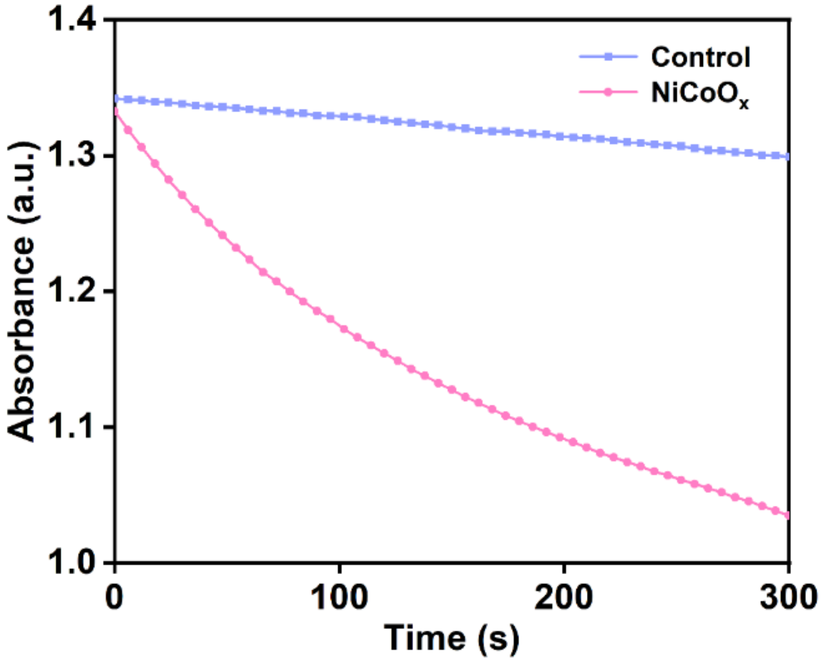

Figure S5. Time-dependent absorbance changes at 420 nm by DPBF probe with 2D $NiCoO_x$.

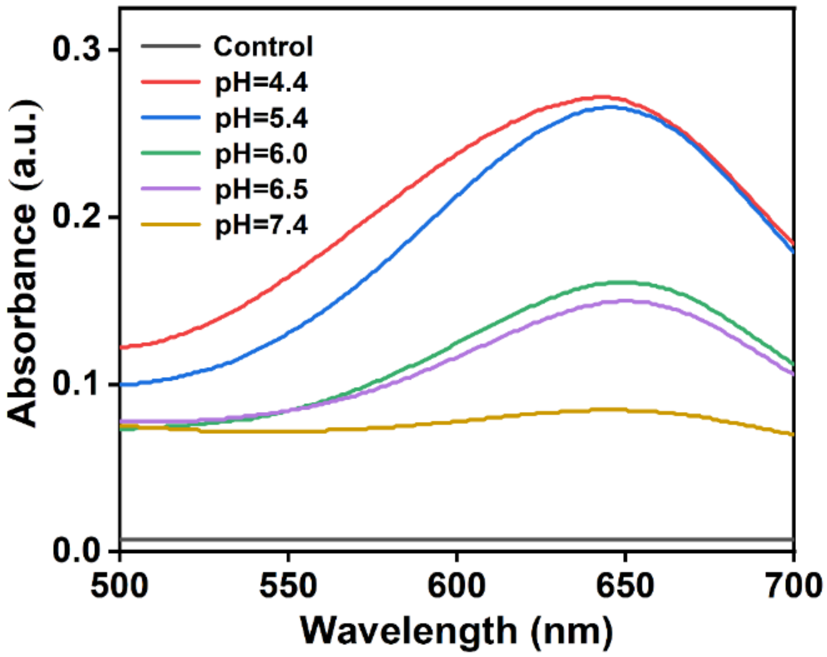

Figure S6. OXD-mimicking activity of 2D $NiCoO_x$ nanosheets tested by TMB probe. The UV-vis absorbance alterations at 652 nm at different pH values (4.4, 5.4, 6, 6.5, 7.4), which is the result of TMB oxidation.

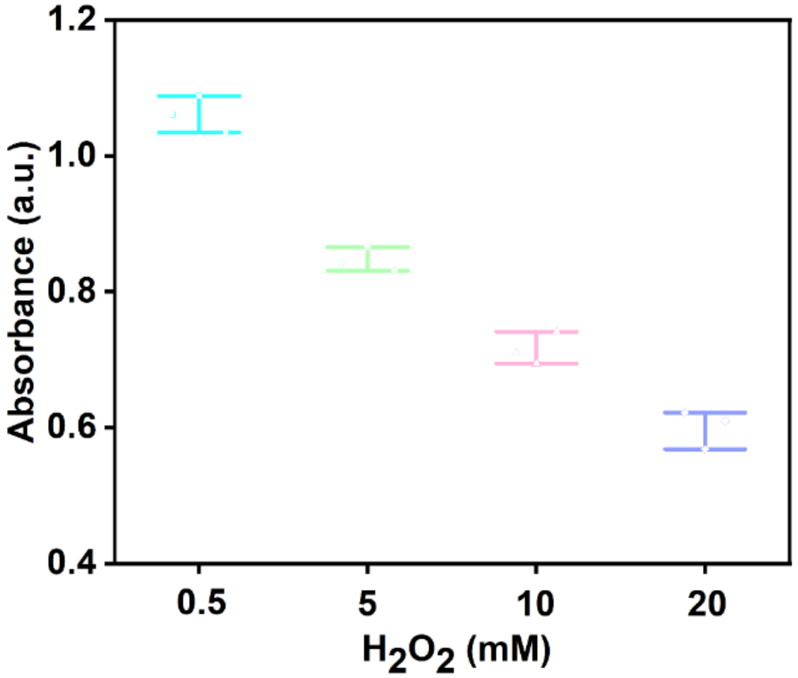

Figure S7. The absorbance decreases at 340 nm with the elevation of H_2O_2 concentration.

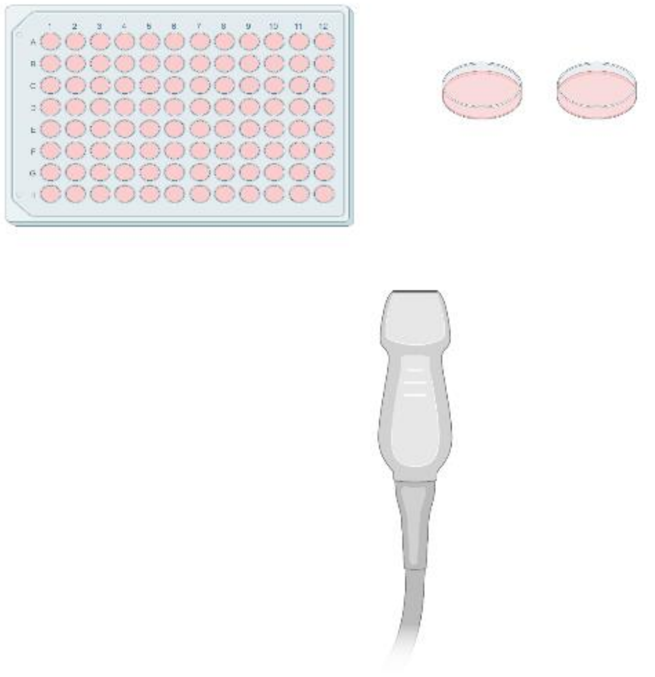

Figure S8. Schematic illustration of the experimental device used with the US radiation for *in vitro* experiment.

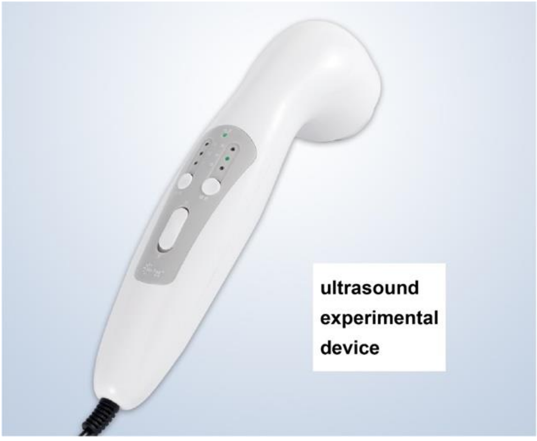

Figure S9. Digital photo of the ultrasound experimental set up used *in vitro* experiments.

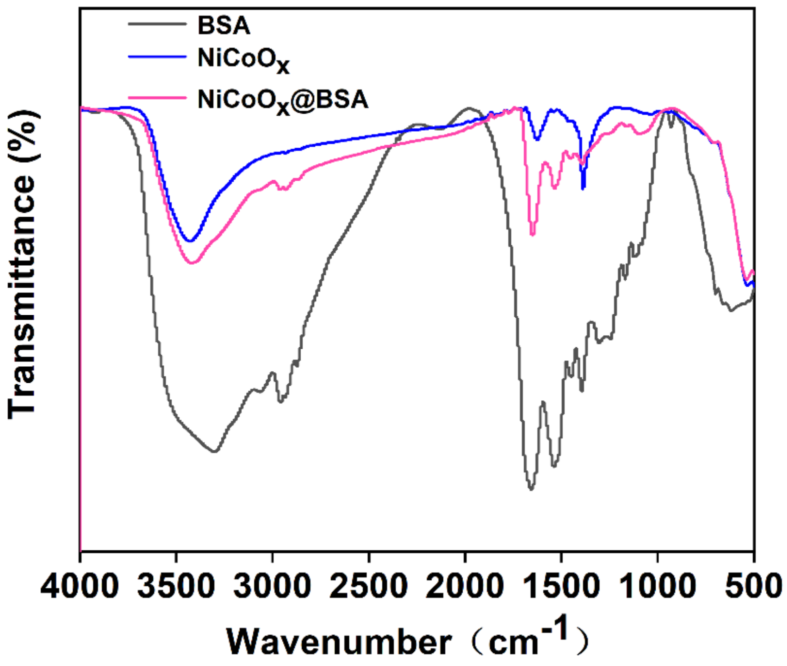

Figure S10. The FTIR of BSA, $NiCoO_x$ and $NiCoO_x$@BSA.

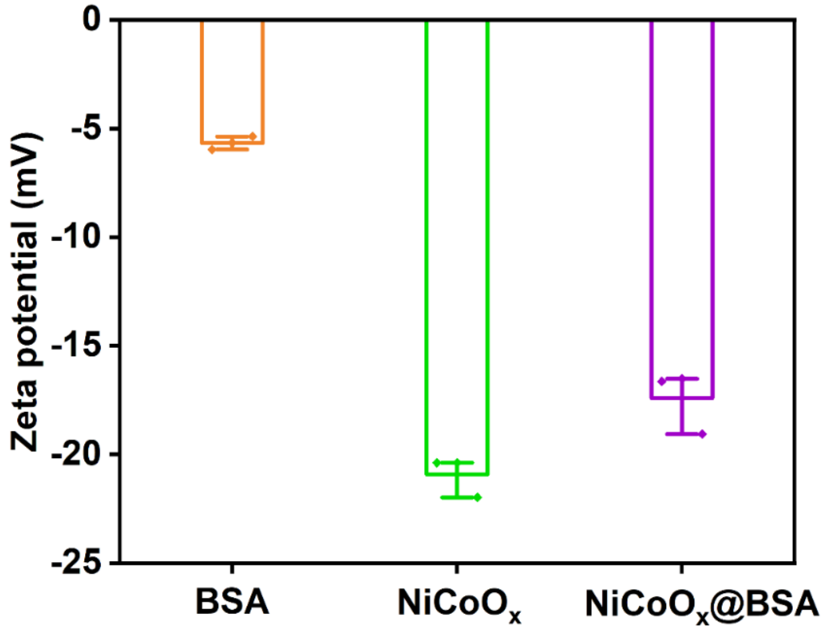

Figure S11. The zeta potential of BSA, $NiCoO_x$ and $NiCoO_x$@BSA.

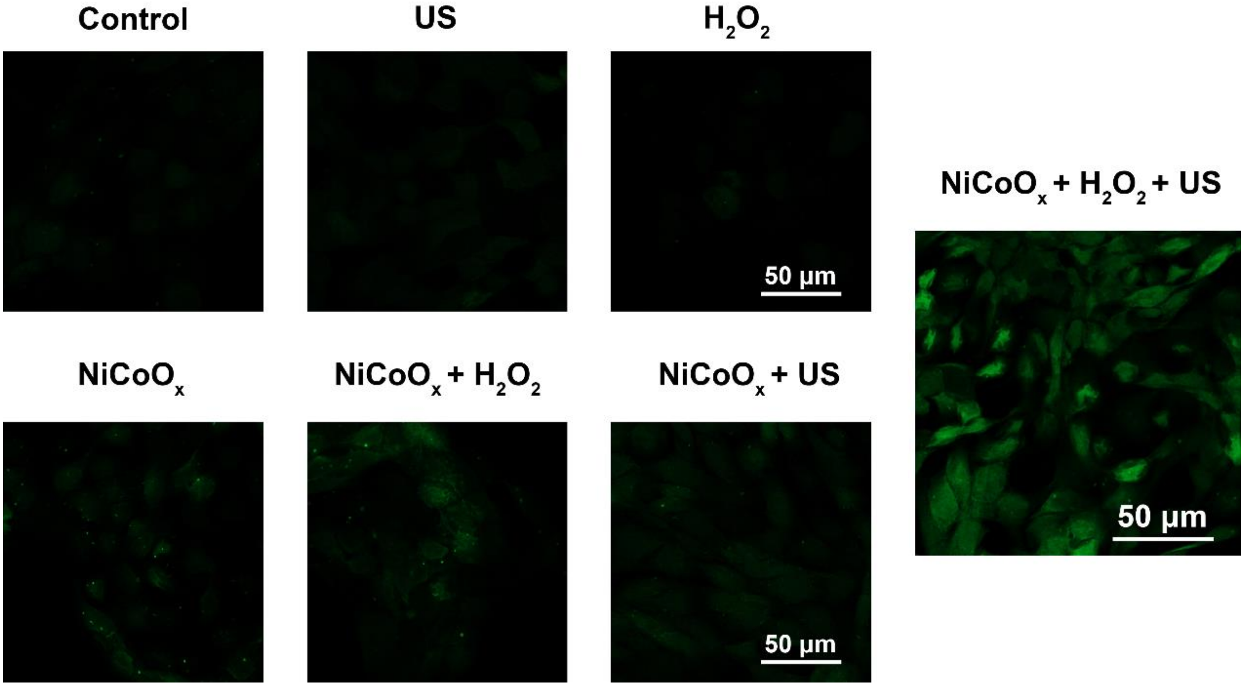

Figure S12. CLSM images of intracellular ROS level after different treatments of 4T1 cells at pH 7.4 (for the groups involving US: 1 MHz; 1.2 W cm^{-2}; 50% duty cycle; and 1 min).

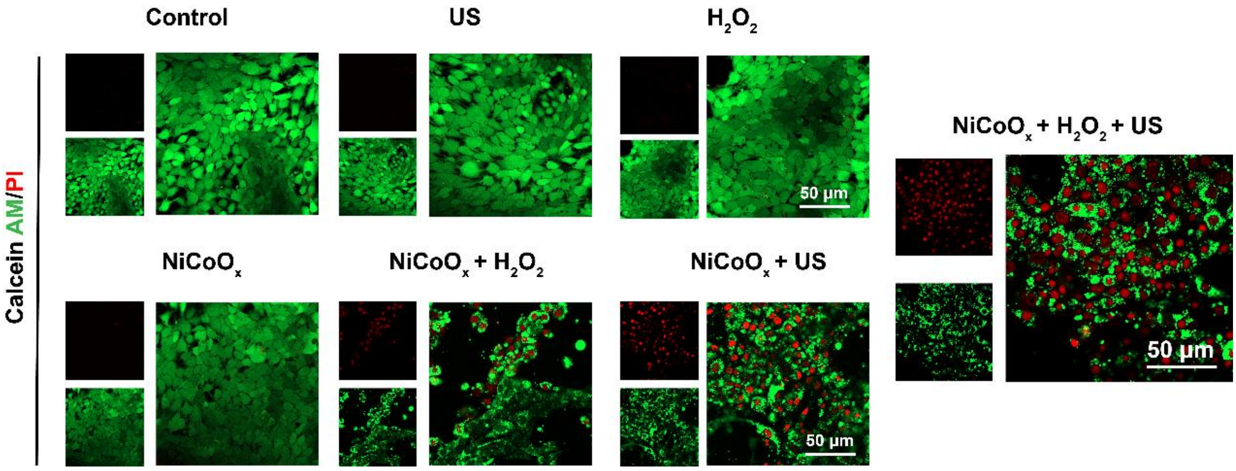

Figure S13. CLSM images of 4T1 tumor cells after different treatments with calcein AM/PI staining at pH 7.4 (for the groups involving US: 1 MHz; 1.2 W cm^{-2}; 50% duty cycle; and 1 min).

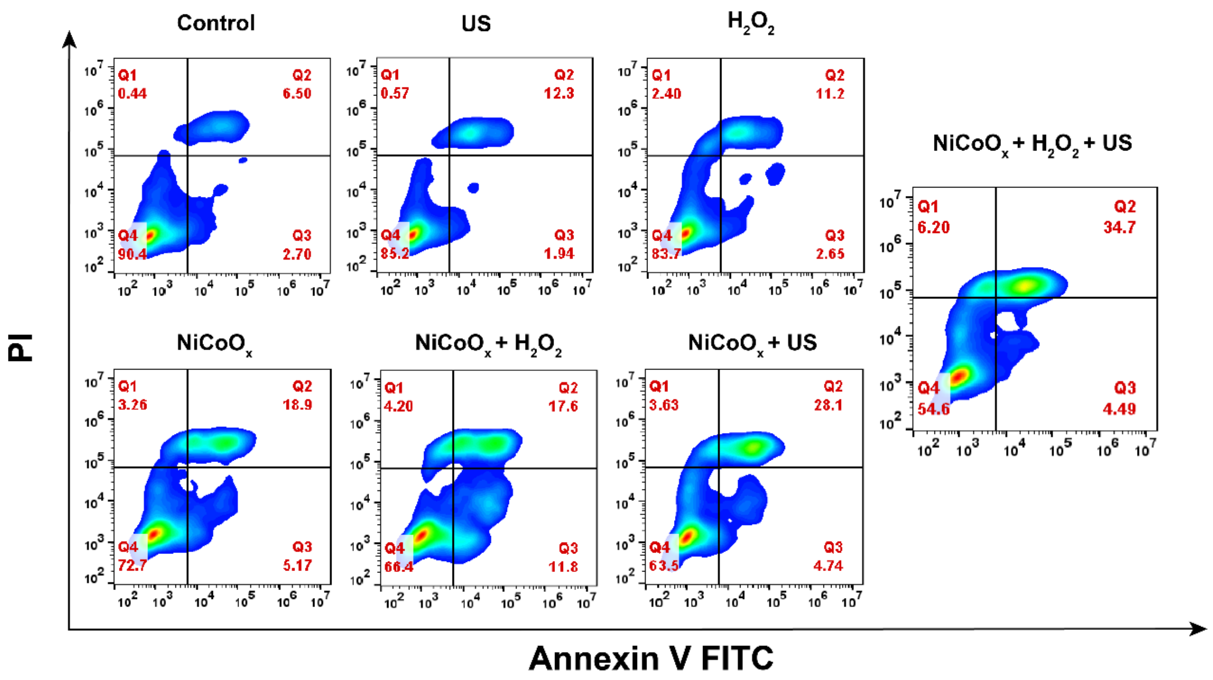

Figure S14. Flow cytometric analysis of 4T1 tumor cells after different treatments at pH 7.4 (for the groups involving US: 1 MHz; 1.2 W cm^{-2}; 50% duty cycle; and 1 min). The relative quantitative analysis of 4T1 tumor cells was handled with various treatments using Annexin-V-FTIC/PI assay at pH 7.4.

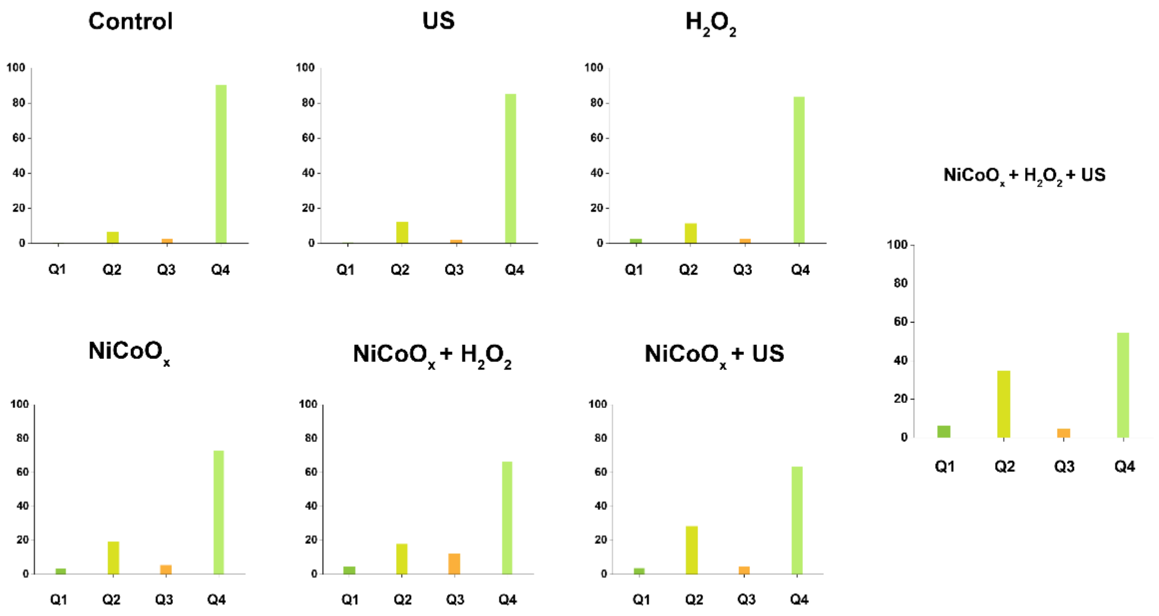

Figure S15. The relative quantitative analysis of 4T1 tumor cells handled with various treatments using Annexin-V-FTIC/PI assay.

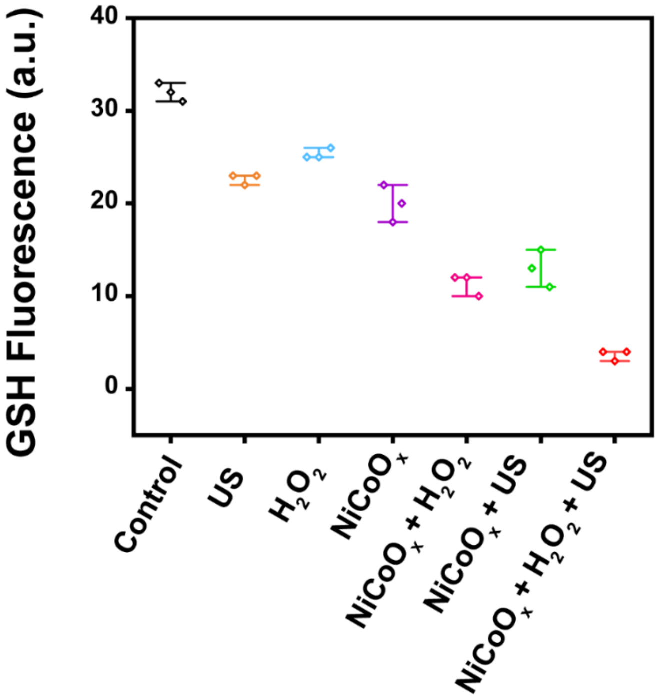

Figure S16. Quantification of intracellular GSH in 4T1 cells with various treatments at pH 6.5.

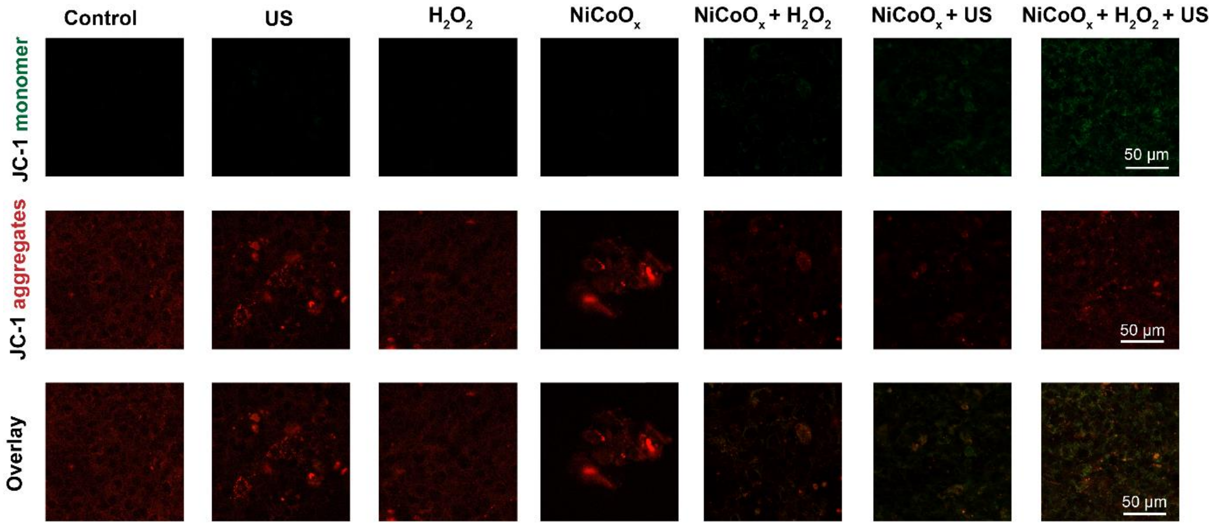

Figure S17. JC-1 assay of 4T1 tumor cells after coincubation with 2D $NiCoO_x$ (100 μg mL^{-1}) by various treatments at pH 7.4 (for the groups involving US: 1 MHz; 1.2 W cm^{-2}; 50% duty cycle; and 1 min).

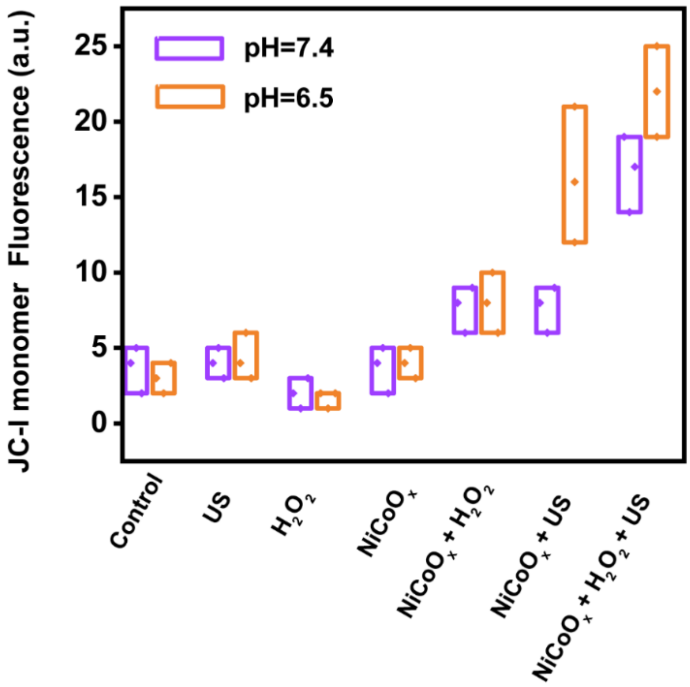

Figure S18. Quantitative JC-1 monomer fluorescence in 4T1 cells treated with various protocols at pH 6.5 and pH 7.4, respectively.

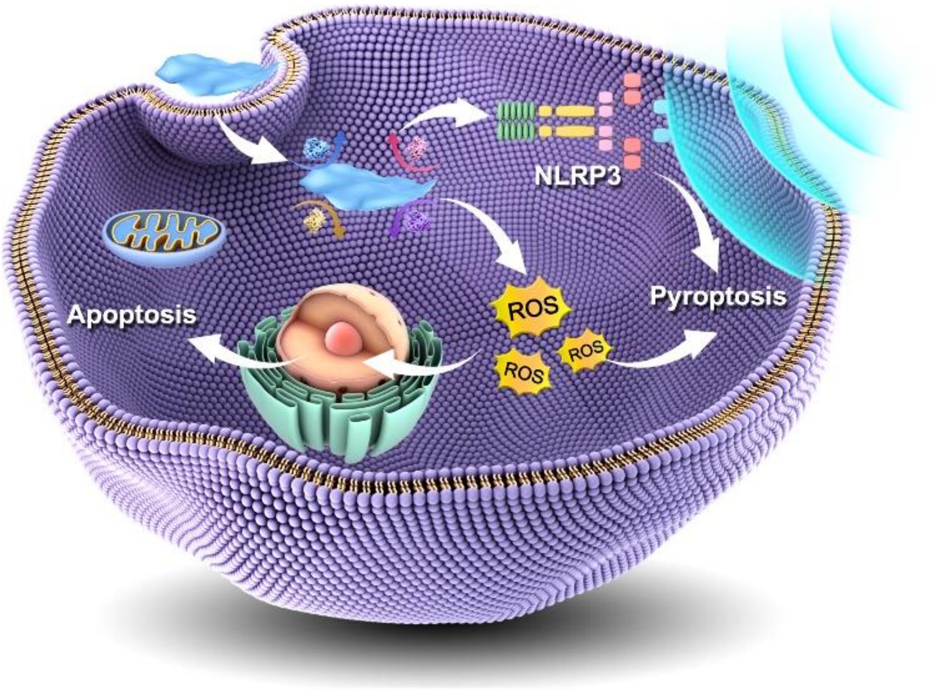

Figure S19. Schematic diagram to illustrate the pyrolysis biomarkers NLRP3 release from 4T1 cells to cause pyroptosis triggered by US-enhanced multienzyme activities of 2D $NiCoO_x$.

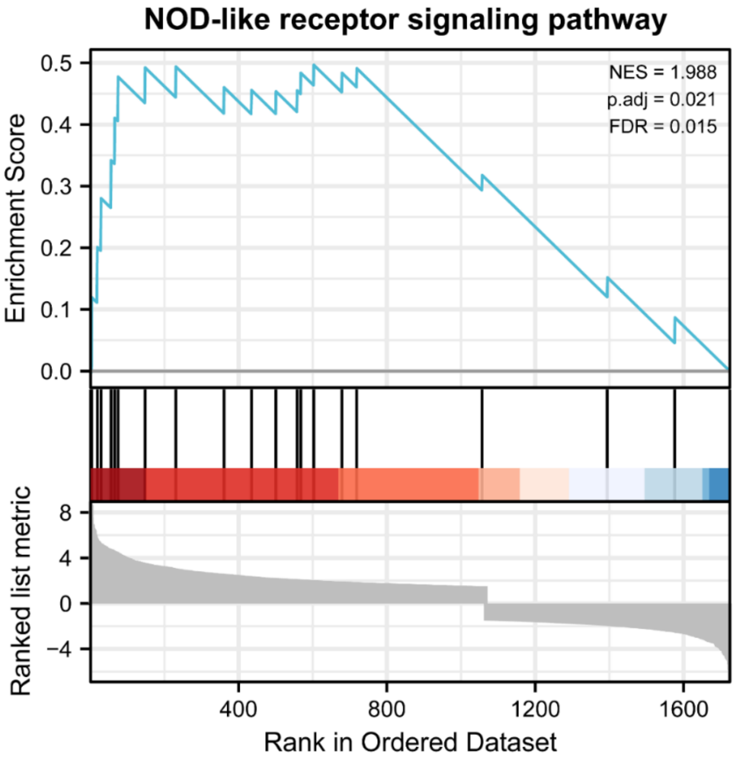

Figure S20. Gene set enrichment analysis (GSEA) of NOD-like receptor signaling pathway.

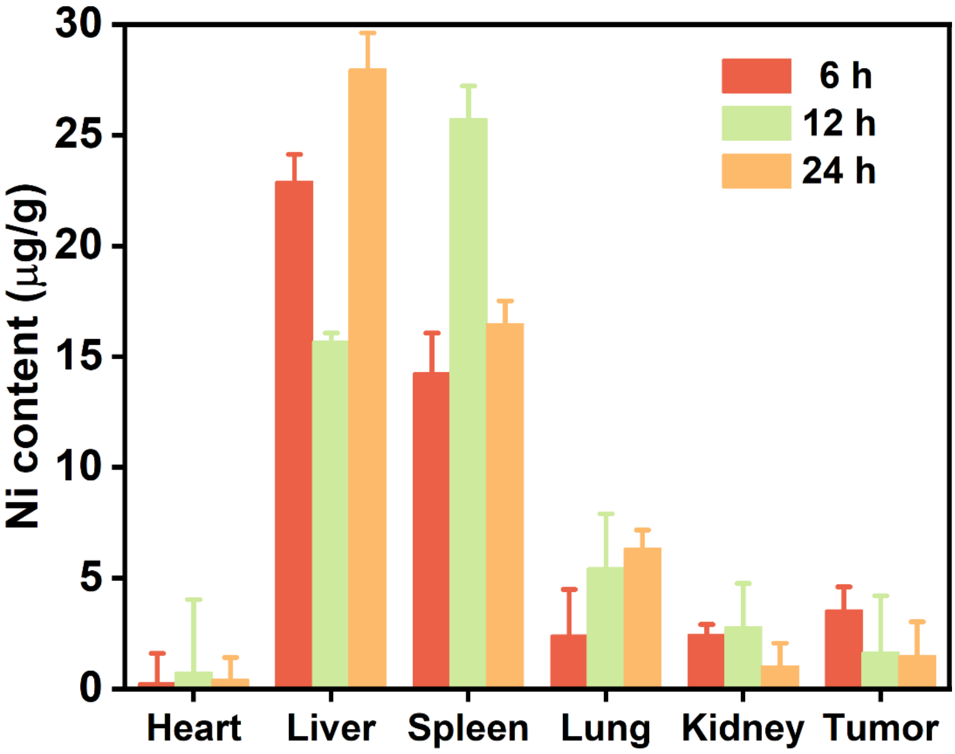

Figure S21. Bio-distribution of Ni content in major organs and tumor by ICP-OES after intravenous injection of $NiCoO_x$.

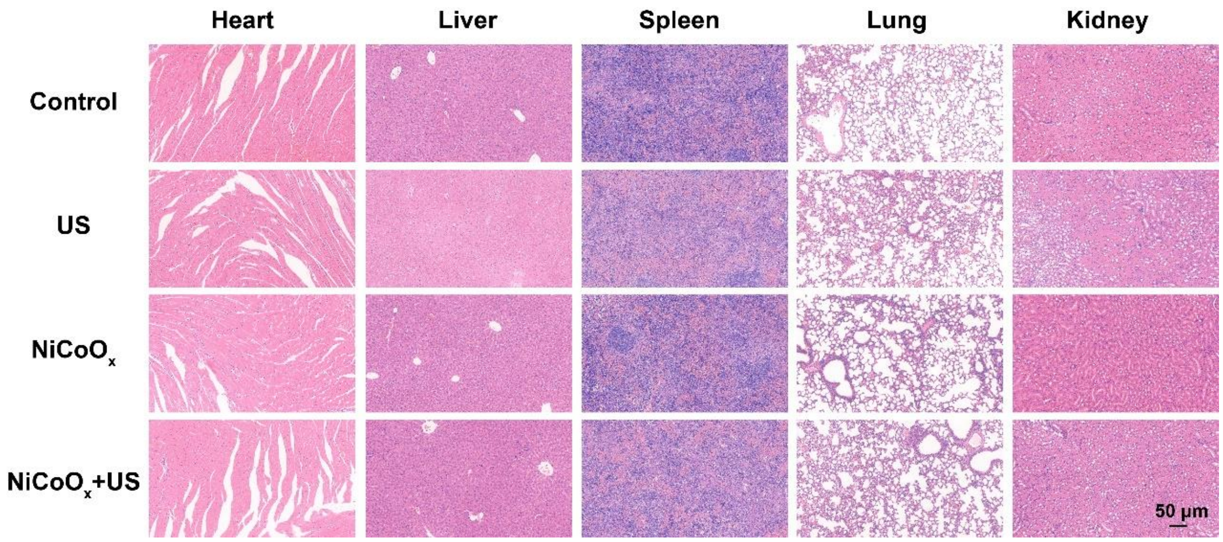

Figure S22. Histological examination photographs of major organs (heart, liver, spleen, lung and kidney) obtained from various treatment groups after therapy.

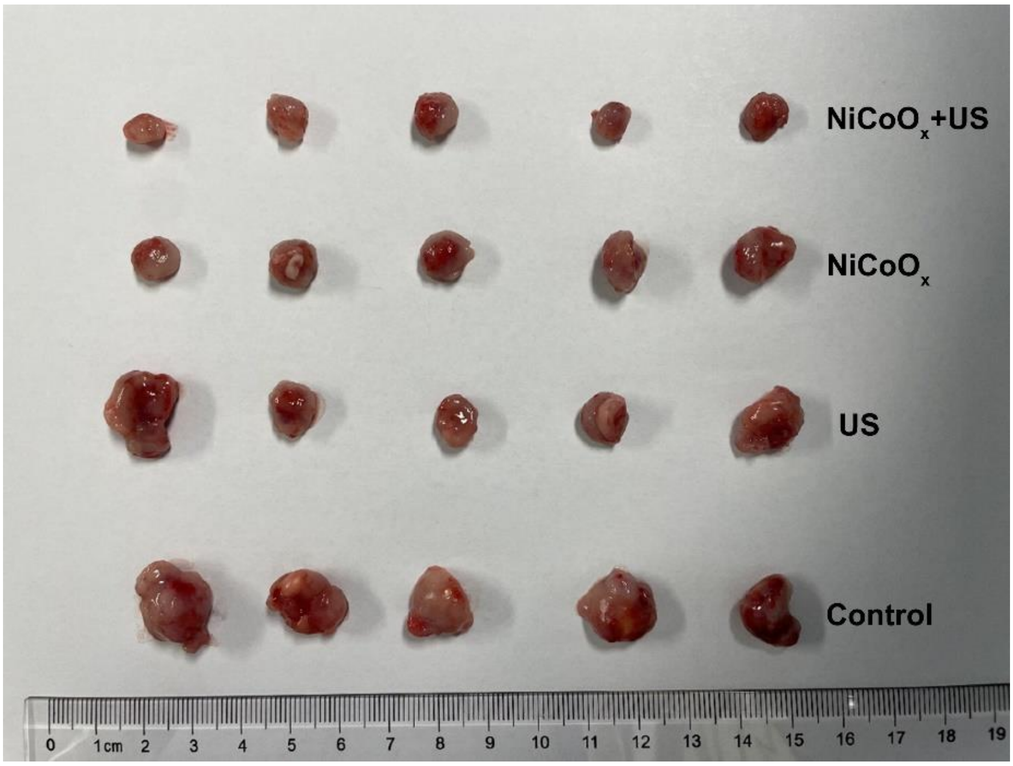

Figure S23. Representative digital photographs of tumor resection from various treatment groups.

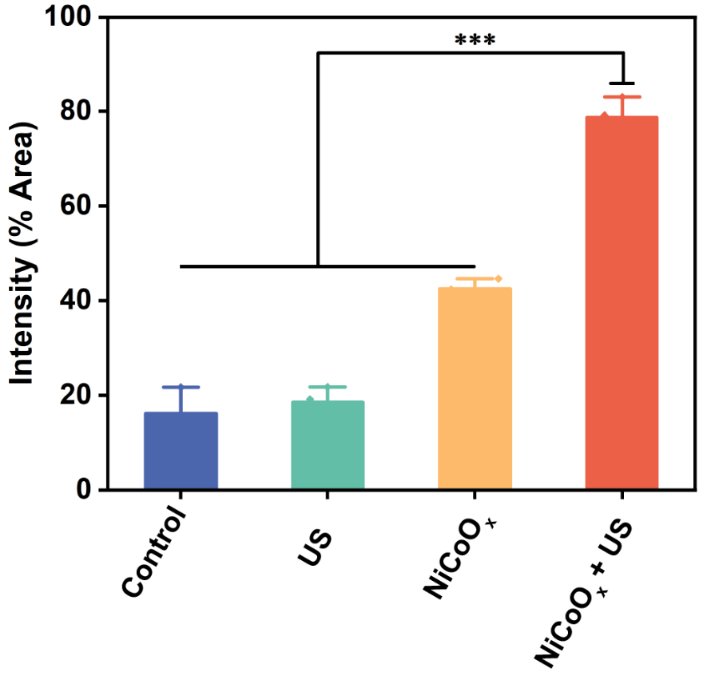

Figure S24. The corresponding quantifications of the immunohistochemical analysis of TUNEL in Figure 6h. The data are shown as the mean ± standard deviation (SD), as calculated using a Student's t-test with $*P < 0.05$, $**P < 0.01$ and $***P < 0.001$.

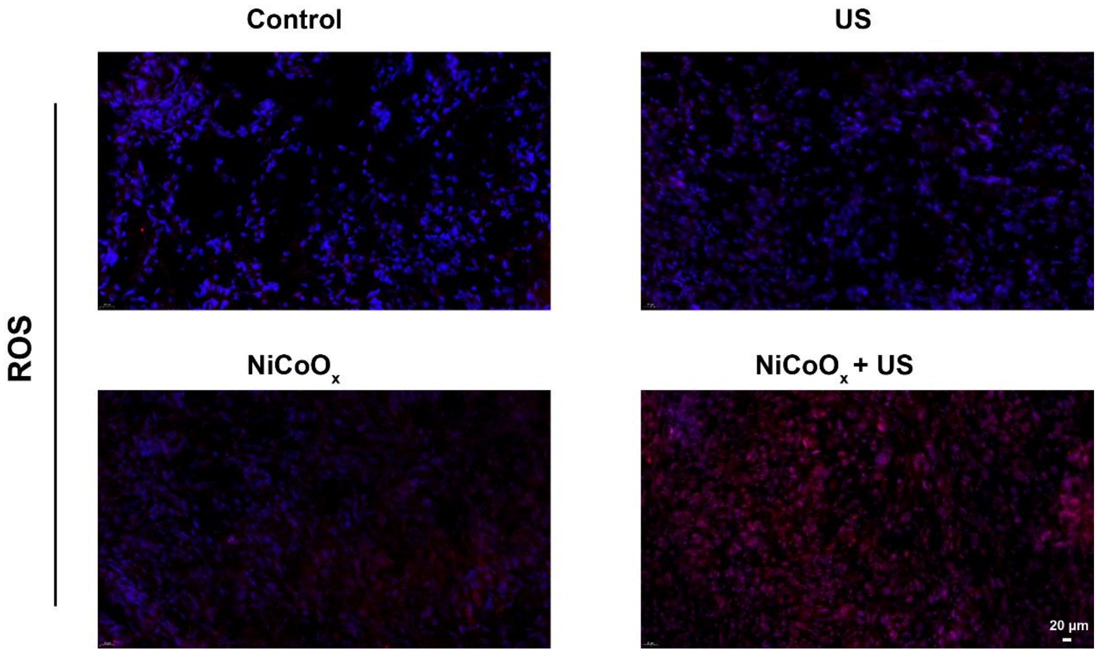

Figure S25. ROS staining images of the tumor slices obtained from the various groups (control, US, $NiCoO_x$, and $NiCoO_x$ + US) of mice.

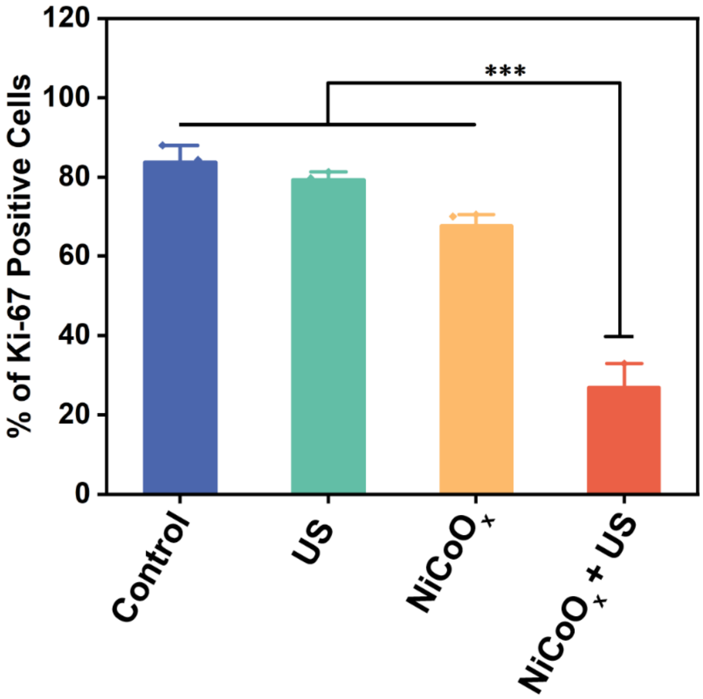

Figure S26. The corresponding quantifications of the immunohistochemical analysis of Ki-67 in Figure 6i. The data are shown as the mean ± standard deviation (SD), as calculated using a Student's t-test with *P < 0.05, **P < 0.01 and ***P < 0.001.

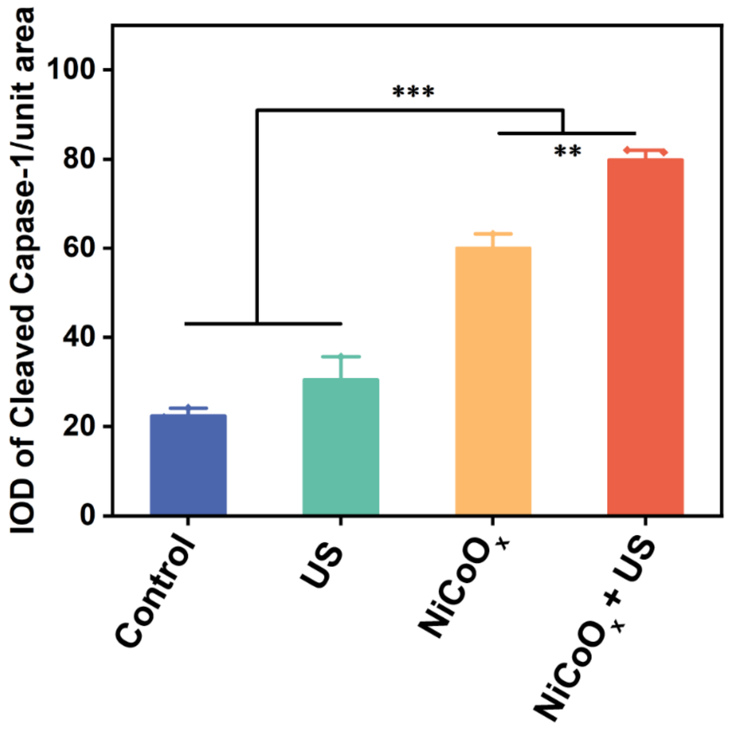

Figure S27. The corresponding quantifications of the immunohistochemical analysis of Cleaved Caspase-1 in Figure 6j. The data are shown as the mean ± standard deviation (SD), as calculated using a Student's t-test with *P < 0.05, **P < 0.01 and ***P < 0.001.

Table S1. Main results of the volcano map.

condition	number	high expression number	low expression number
Total number of ID	13393		
|Log2(FC)|＞1 & p.adj＜0.05	3399	1917	1482
|Log2(FC)|＞1.5 & p.adj＜0.05	1786	1104	682
|Log2(FC)|＞2 & p.adj＜0.05	966	642	324

Parameter: LogFC > 1.5 & p value < 0.05 in the figure.

Table S2. The detailed information about Gene Ontology (GO) analysis of the $NiCoO_x$ + US group and control group.

Ontology	ID	Description	Gene Ratio	P value
BP	GO:0009615	response to virus	56/1037	2.83E-22
BP	GO:0051607	defense response to virus	46/1037	4.2148E-18
BP	GO:0051090	regulation of DNA−binding transcription factor activity	60/1037	9.151E-17
CC	GO:0020003	symbiont-containing vacuole	6/1030	1.5738E-05
CC	GO: 0005667	transcription factor complex	38/1030	2.4076E-05
CC	GO: 0065010	extracellular membrane-bounded organelle	6/1030	3.8902E-05
MF	GO: 0001228	DNA-binding transcription activator activity, RNA polymerase II-specific	53/1028	8.3219E-11
MF	GO:0048018	receptor ligand activity	56/1028	1.8704E-11
MF	GO:0005125	cytokine activity	34/1028	1.9162E-10
KEGG	mmu04668	TNF signaling pathway	32/470	1.2671E-15
KEGG	mmu05164	Influenza A	34/470	1.5897E-11
KEGG	mmu04657	IL-17 signaling pathway	23/470	1.7796E-10

Table S3. The detailed information about KEGG analysis of the $NiCoO_x$ + US group and control group.

Pathway ID	Pathway	P value	gene
GO: 0045089	positive regulation of innate immune response	3.7772E-12	H2-T23<br>Irgm1<br>Irf1<br>Acod1<br>Lgals9<br>Mmp12<br>Nfkbia<br>Prkce<br>Trim30a<br>Tnf<br>Tnfaip3<br>Tlr2<br>Irf7<br>Irgm2<br>Crtam<br>Ikbke<br>Tnip1<br>Rsad2<br>Zbp1<br>Pycard<br>Trim15<br>Ifih1<br>Zc3hav1<br>Parp9<br>Dhx58<br>Ankrd17<br>Havcr2<br>Ripk2<br>Nr1d1<br>Gbp5<br>Ddx58<br>Ddx60<br>Klre1<br>Nod2<br>Tnip3<br>Nlrc5<br>Mndal<br>Epg5
GO: 0045088	regulation of innate immune response	1.0267E-12	H2-T23<br>Irgm1<br>Irf1<br>Acod1<br>Lgals9<br>Mmp12<br>Nfkbia<br>Prkce

			Trim30a<br>Tnf<br>Tnfaip3<br>Tlr2<br>Irf7<br>Irgm2<br>Crtam<br>Adar<br>Ikbke<br>Tnip1<br>Rsad2<br>Zbp1<br>Dusp10<br>Pycard<br>Trim15<br>Ifih1<br>Zc3hav1<br>Parp9<br>Dhx58<br>Ankrd17<br>Clec2d<br>Havcr2<br>Ripk2<br>Nr1d1<br>Gbp5<br>Ddx58<br>Trafd1<br>Ddx60<br>Klre1<br>Nod2<br>Tnip3<br>Nlrc5<br>Parp14<br>Mndal<br>Epg5
mmu04621	NOD-like receptor signaling pathway	5.3239E-10	Birc3<br>Casp4<br>Gbp2b<br>Gbp2<br>Cxcl1<br>Irf9<br>Itpr2<br>Jun<br>Nfkb1<br>Nfkbia<br>Nfkbib<br>Ccl2<br>Ccl5<br>Cxcl2<br>Stat1

			Stat2<br>Tank<br>Tnf<br>Tnfaip3<br>Traf6<br>Irf7<br>Tyk2<br>Gbp3<br>Ikbke<br>Nampt<br>Pycard<br>Ripk2<br>Nlrp3<br>Gbp5<br>Gbp7<br>Oas3<br>Oas2<br>Oas1a<br>Nod2<br>Rnf31<br>Cxcl3
